# Hydrogel-based delivery of extracellular vesicles for skin wound healing: design strategies, therapeutic mechanisms, and translational perspectives

**DOI:** 10.3389/fbioe.2026.1976532

**Published:** 2026-10-16

**Authors:** Yuqiu Jin, Jun Lu, Jingping Wu, Yi Zuo, Huilan Zheng, Fengrui Cheng

**Affiliations:** 1 Department of Medical Cosmetology, Hospital of Chengdu University of Traditional Chinese Medicine, Chengdu, China; 2 State Key Laboratory of Southwestern Chinese Medicine Resources, School of Pharmacy, Chengdu University of Traditional Chinese Medicine, Chengdu, China

**Keywords:** clinical translation, drug delivery, extracellular vesicles, hydrogel-EV systems, hydrogels, wound healing

## Abstract

Skin wound healing remains a formidable clinical challenge. Extracellular vesicles (EVs) have attracted considerable attention as therapeutic candidates for wound repair, given their intrinsic capacities to modulate inflammation, promote angiogenesis, and stimulate tissue regeneration. Hydrogels, with favorable biocompatibility, tunable degradation, and sustained-release properties, serve as candidate EV delivery platforms. This review critically examines the landscape of hydrogel-mediated EV delivery for skin wound repair, progressing from EV sources and engineering strategies, through hydrogel design principles and crosslinking modalities, to the modes of EV-hydrogel integration and the multi-dimensional mechanisms including immunomodulation, angiogenesis, extracellular matrix remodeling, and cell proliferation that drive the healing process. Recent advances across acute, diabetic chronic, and burn wounds are evaluated, together with stimuli-responsive hydrogels capable of sensing wound microenvironmental cues such as reactive oxygen species, pH, and matrix metalloproteinase activity to enable microenvironment-responsive EV release, representing a transition from passive delivery toward more adaptive and controlled therapeutic strategies. Nevertheless, the current evidence is derived predominantly from *in vitro* and small-animal studies and is constrained by heterogeneous EV preparations, insufficient standardization of manufacturing and quality control, and limited clinical validation. Finally, critical challenges including production standardization, scalable manufacturing, and regulatory barriers to clinical translation are analyzed, and future directions for advancing hydrogel-EV systems toward clinical translation are discussed, aiming to provide insights and perspectives for further fundamental research and clinical translation of hydrogel-EV systems in skin wound healing.

## 1 Introduction

As the largest organ of the human body, the skin constitutes the first barrier against environmental insults. Skin wounds, spanning surgical incisions, chronic refractory diabetic ulcers, and burns, are among the most prevalent conditions in clinical practice. Of these, chronic ulcers, diabetic wounds, and deep burns pose the greatest therapeutic challenge, marked by refractory healing, elevated infection risk, substantial economic burden, and severely compromised quality of life (Monaghan et al., 2023; Riaz et al., 2025). Notably, diabetes-related wounds frequently progress to amputation and other severe outcomes, significantly increasing disability and mortality rates (Armstrong et al., 2023; Lehn et al., 2026; Ma et al., 2026). Confronted with this formidable clinical challenge, the exploration of more effective wound repair strategies has become an urgent endeavor in regenerative medicine.

Conventional wound management approaches, such as debridement, autologous skin grafting, and topical growth factor administration, have improved clinical outcomes to a certain extent. However, their ability to modulate the complex pathophysiological cascades involved in wound healing, including excessive inflammation, impaired angiogenesis, and dysregulated extracellular matrix (ECM) remodeling, remains limited, and these approaches carry risks of adverse effects such as abnormal skin pigmentation and hypertrophic scarring (Mullin et al., 2024; Zhang et al., 2026a). These limitations have motivated the exploration of alternative therapeutic strategies for wound repair. In recent years, EVs have been increasingly investigated as cell-free therapeutic agents, providing new opportunities for wound repair (Garima et al., 2023; Ding L. et al., 2025). EVs are nanoscale lipid bilayer vesicles secreted by cells, carrying diverse bioactive molecules including proteins, nucleic acids, lipids, and metabolites. As important mediators of intercellular communication, they participate extensively in wound healing, immune regulation, development, and tissue homeostasis (Aimaletdinov and Gomzikova, 2022; Takahashi and Takakura, 2023).

EVs can be derived from virtually all cell types and are ubiquitously present in biological fluids such as blood, urine, saliva, and milk (Luo et al., 2024; Nunes et al., 2025). EVs from different cellular sources exhibit distinct biological characteristics and functional profiles (Quiñones et al., 2024). Furthermore, EVs can be engineered through genetic, chemical, or physical approaches to enhance their targeting capacity and therapeutic efficacy, thereby extending their application boundaries (Hade et al., 2022). Nevertheless, both native and engineered EVs face common bottlenecks *in vivo*, including short half-life, susceptibility to nuclease degradation, rapid clearance by the mononuclear phagocyte system, and poor local retention at the wound site, all of which severely constrain their therapeutic potential. Given the diversity of vesicular systems investigated in wound healing, this review primarily focuses on naturally released EVs investigated for skin wound repair, particularly mammalian cell-derived EVs, while also including vesicles derived from bacterial and plant sources. Apoptotic extracellular vesicles (ApoEVs) are included as a distinct EV population, whereas artificially generated biomimetic nanovesicles are discussed separately as EV-related vesicular systems rather than naturally secreted EVs.

To address these delivery barriers, biomaterial-based hydrogels have emerged as an attractive carrier platform for EVs, owing to their unique physicochemical properties. Hydrogels are three-dimensional (3D) networks formed by physical or chemical crosslinking of hydrophilic polymers, offering high water content, favorable tissue compatibility, and tunable degradation properties. They can mimic the microenvironmental characteristics of native ECM, establish a protective reservoir at the wound site, effectively withstand exudate washing, and retard enzymatic degradation, thereby enabling controlled and sustained release of EVs with prolonged bioactivity (Hosseini et al., 2025). More importantly, the integration of EVs with hydrogels is not a simple physical mixture. The mode of EV-hydrogel combination directly influences the release kinetics and functional retention of EVs, while the composite system drives the healing process through multi-dimensional mechanisms including modulation of the wound inflammatory microenvironment, promotion of functional angiogenesis, acceleration of ECM remodeling, and enhancement of reparative cell proliferation and migration (Chen Y. et al., 2023). Building on these features, stimuli-responsive hydrogels can sense pathological signals in the wound microenvironment, such as reduced pH, accumulated reactive oxygen species (ROS), and overexpressed matrix metalloproteinases (MMPs), and enable microenvironment-responsive and controlled release of EVs (Zeng et al., 2025). These multiple advantages have placed hydrogel-EV composite systems among the actively investigated approaches in current wound repair research.

This review provides a comprehensive examination of hydrogel-mediated EV delivery for skin wound repair, progressing from EV source diversity and engineering strategies, through hydrogel design principles and stimuli-responsive systems, to the modes of EV-hydrogel integration and their underlying pro-repair mechanisms. Application progress across acute, diabetic chronic, burn wounds and other wound types is then evaluated, followed by a critical discussion of the major bottlenecks impeding clinical translation, aiming to provide an overview for both fundamental investigation and clinical development in this field. Unlike previous reviews that mainly summarize EV engineering strategies or hydrogel classifications, this review proposes a pathology-oriented framework for designing hydrogel-EV systems according to the distinct microenvironmental characteristics of different wound types, thereby providing a conceptual basis for pathology-oriented wound repair.

## 2 Literature search and study selection

A structured literature search was conducted in PubMed and the Web of Science Core Collection (WoSCC) from database inception to 25 September 2026. The search combined terms related to extracellular vesicles (“extracellular vesicle*” OR “exosome*”), hydrogels (“hydrogel*”), and skin wound healing (“skin wound*,” “wound healing,” “diabetic wound*,” “chronic wound*,” “infected wound*,” “burn wound*,” “surgical wound*,” and “skin regeneration”). Original experimental studies were considered core evidence when they investigated EVs or clearly defined EV-related vesicular systems delivered using a hydrogel-based platform and evaluated the resulting system in cutaneous wound healing or skin regeneration. Studies without an EV-based vesicular component or hydrogel delivery system, studies unrelated to cutaneous wound healing or skin regeneration, non-original publications, duplicate reports, and studies with insufficient information for assessment were excluded from the core evidence set. Additional references were used as background evidence for EV characterization, hydrogel design, wound pathophysiology, stimuli-responsive hydrogels, manufacturing, safety, and regulatory considerations. Detailed database-specific search strategies and study selection criteria are provided in the Supplementary Material.

## 3 EVs: sources, characterization, and engineering

EVs have attracted broad attention in wound repair research owing to their intrinsic biocompatibility, low immunogenicity, diverse cellular origins, and capacity to carry a broad repertoire of bioactive cargos, including proteins, mRNA, miRNA, lncRNA, and circRNA, that regulate recipient cell function through intercellular communication. These attributes establish EVs as both natural therapeutic agents and versatile platforms for engineering modification, the foundations of which are examined in the following sections (Figure 1).

**FIGURE 1:**
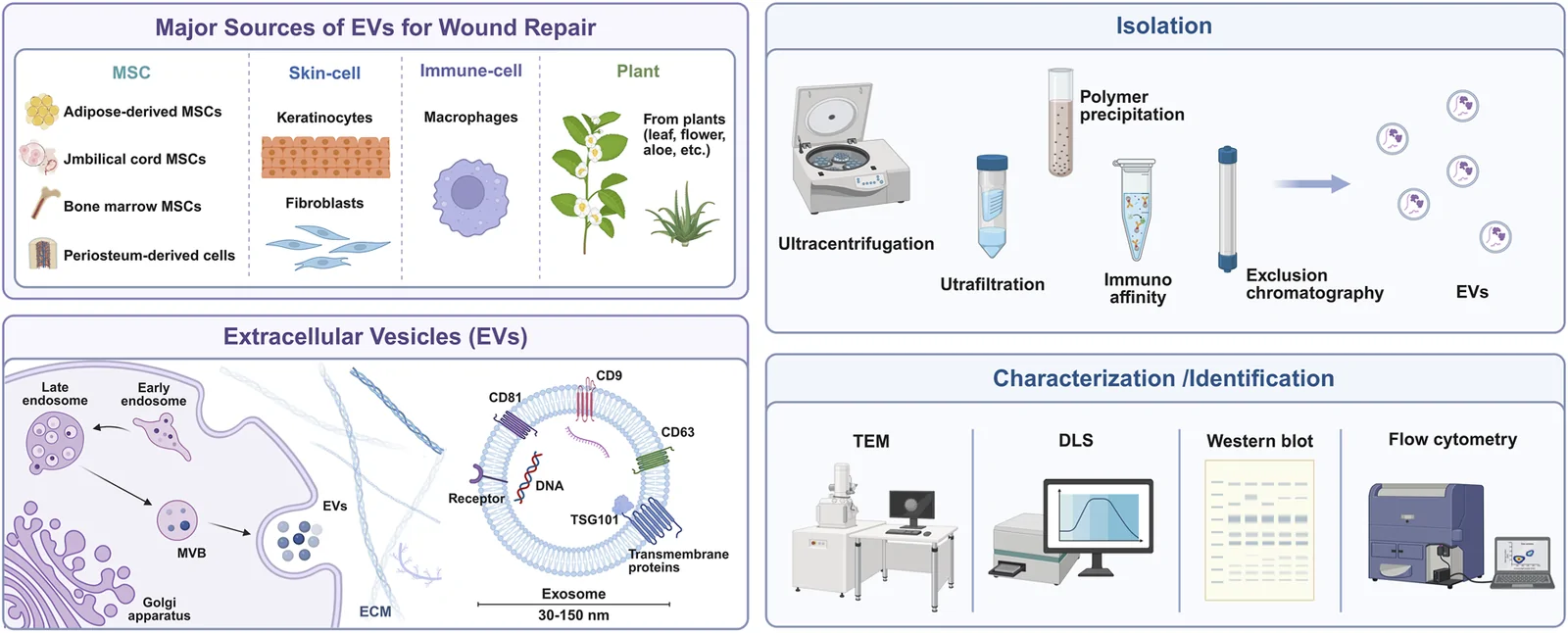
The major sources, isolation strategies and identification methods of extracellular vesicles (EVs). EVs can be derived from multiple sources, including mesenchymal stem cells (MSCs), immune cells, skin-resident cells and plant cells. Representative isolation methods include ultracentrifugation (UC), precipitation, ultrafiltration (UF), size-exclusion chromatography (SEC), and immunoaffinity capture (IAC). EV characterization includes morphological and particle-size analyses using transmission electron microscopy (TEM) and dynamic light scattering (DLS), together with the assessment of EV-associated positive and negative protein markers by Western blotting or flow cytometry.

### 3.1 Sources and classifications of EVs

EVs employed in wound repair derive from a broad spectrum of cell types, each imparting a distinct functional bias. Among them, mesenchymal stem cell (MSC)-derived EVs, whether sourced from adipose tissue, umbilical cord, bone marrow, or periosteum, represent the most intensively studied category owing to their anti-inflammatory, pro-angiogenic, and pro-epithelialization capacities (Liao et al., 2022; Neupane et al., 2023; Wang et al., 2023; Kaiser et al., 2024; Chen X. et al., 2025; Li R. et al., 2025). Skin-resident cell-derived EVs serve functionally specialized roles, with keratinocyte-derived EVs driving re-epithelialization, fibroblast-derived EVs facilitating matrix remodeling, and human umbilical vein endothelial cell (HUVEC)-derived EVs being widely utilized for their pro-angiogenic properties (Glady et al., 2021; Parvanian et al., 2025). Immune cell-derived EVs, particularly molybdenum-induced macrophage-derived EVs (Mo-EVs), predominantly mediate inflammatory regulation (Park et al., 2024). Beyond mammalian sources, plant-derived EVs from quercetin, hydrocotyle chinensis, microalgae, and aloe, together with royal jelly-derived EVs, have been investigated for chronic wound repair, owing to their low cost, high stability, and low immunogenicity (Kim et al., 2021; Tan et al., 2023).

From the perspective of biogenesis pathway, EVs can be classified into four major subtypes: exosomes, microvesicles, apoptotic vesicles, and migrasomes, each with distinct features in size, origin, cargo, and function (Liu S. et al., 2022). Exosomes originate from the endosomal system. The plasma membrane invaginates to form early endosomes, which mature into multivesicular bodies that subsequently fuse with the plasma membrane to release exosomes with a diameter of approximately 30–150 nm (Sheng et al., 2025). Their canonical markers include the tetraspanin family (CD9, CD63, CD81), TSG101, and Hsp70 (Dixson et al., 2023). The membrane structure of exosomes resembles that of the plasma membrane, and their cargoes are selectively packaged and regulated by the physiological state of parental cells. Microvesicles are formed by direct outward budding and shedding of the plasma membrane, generally 100–1,000 nm in diameter, and similarly carry diverse bioactive molecules mediating both local and distant signaling (Zhu et al., 2021). Apoptotic vesicles are larger vesicles produced during programmed cell apoptosis, mainly cleared by macrophage phagocytosis, and participate in immune regulation through the transmission of apoptotic signals (Zou et al., 2023). Migrasomes form at the tips or intersections of retraction fibers at the rear of migrating cells and are released via migracytosis, reaching up to 3 μm in diameter. Their cargos are released after transit through migrasomes and participate in spatiotemporally specific signal transmission, mitochondrial quality control, and tissue remodeling (Xu et al., 2023). Among these subtypes, exosomes have been extensively investigated in wound healing applications due to their favorable biocompatibility, low immunogenicity, circulatory stability, and engineering modifiability (Ye et al., 2023). In this review, EVs are used as the overarching term for naturally released extracellular vesicles, while specific terms such as small EVs (sEVs) and apoptotic extracellular vesicles (ApoEVs) are retained where justified by the original studies. Engineered biomimetic nanovesicles are discussed separately as EV-related vesicular systems because their artificial production distinguishes them from naturally secreted EVs.

In brief, the diversity of EV sources and the functional heterogeneity of EV subtypes provide multiple potential options for wound repair. Cellular origin determines the bioactivity profile of EVs, while biogenesis pathway dictates their structural features and cargo repertoire. Rational selection of both is an important prerequisite for designing hydrogel-EV composite systems.

### 3.2 Isolation, identification and quality control of EVs

The stable acquisition of high-purity, high-activity EVs is a prerequisite for wound repair research. EV isolation methods can be categorized into five types based on underlying principles: ultracentrifugation (sedimentation coefficient), precipitation (solubility), ultrafiltration (particle size), immunoaffinity capture (surface marker affinity), and size-exclusion chromatography (SEC; hydrodynamic size), each with distinct trade-offs in purity, yield, equipment requirements, and impact on vesicle structure (Aliakbari et al., 2024).

Ultracentrifugation is currently the most widely used method, employing high centrifugal force to achieve stepwise sedimentation of differently sized vesicles. It is often combined with differential centrifugation or sucrose/iodixanol density gradient centrifugation to improve purity (Onódi et al., 2018). Its advantages lie in high yield and suitability for large-volume samples, while its limitations include long processing time, heavy equipment dependence, and potential co-precipitation of non-vesicular impurities. The precipitation method reduces vesicle solubility by adding polymers such as polyethylene glycol or adjusting ionic strength to promote precipitation from solution, offering operational simplicity and low cost but relatively limited purity (Gardiner et al., 2016). Ultrafiltration employs membranes with specific pore sizes to selectively retain vesicles under mild conditions with high recovery rates and can be combined with other methods such as SEC to further enhance purity (Gao et al., 2022). Immunoaffinity capture achieves targeted enrichment based on high-affinity binding of antibodies, aptamers, or lectins to vesicle surface-specific antigens such as CD9, CD63, CD81, and L1CAM (Chen M. et al., 2024). This method provides high specificity and can be integrated with magnetic beads for automation, though it incurs higher costs and depends on high-quality antibodies and established markers. SEC separates particles according to hydrodynamic size differences, wherein small-molecule impurities are retained within the gel pores while larger vesicles elute first, effectively removing non-vesicular proteins such as lipoproteins with minimal structural damage.

Methods for the identification of EVs are shown below. Morphological characterization primarily relies on transmission electron microscopy to confirm the typical nano-scale lipid bilayer vesicle morphology (Bonavina et al., 2024). Size characterization employs nanoparticle tracking analysis or dynamic light scattering with subtype-specific size reference ranges (Chen J. W. et al., 2023). Molecular marker characterization is performed using Western blot or flow cytometry to detect tetraspanins (CD9, CD63 and CD81), which are commonly assessed EV-associated markers, together with negative protein markers such as calnexin (Tkach and Théry, 2016; Fan et al., 2023; Zhang Q. et al., 2025). Establishing standardized EV preparation and characterization workflows is a critical prerequisite for advancing this field from the laboratory to the clinic.

Since different isolation methods yield EV preparations with distinct physicochemical characteristics, comprehensive quality control is essential before therapeutic application. According to the MISEV2023 guidelines and recent quality control frameworks for EV therapeutics, critical quality attributes include identity, purity, concentration, potency, sterility, endotoxin content, stability, aggregation, and batch-to-batch consistency (Welsh et al., 2024; Moon et al., 2026).

Isolation methods substantially influence the critical quality attributes of EV preparations (Table 1). Differential ultracentrifugation generally provides relatively high recovery but may induce vesicle aggregation and co-isolation of soluble proteins due to prolonged high-speed centrifugation, thereby compromising EV purity and biological activity (Gardiner et al., 2016). In contrast, size-exclusion chromatography (SEC) effectively separates EVs from soluble proteins and lipoproteins according to hydrodynamic size while largely preserving vesicle integrity, making it an approach for obtaining relatively high-purity EVs (Böing et al., 2014). Ultrafiltration, which separates vesicles based on particle size using membranes with defined molecular weight cut-offs, offers high recovery under relatively mild processing conditions and better preserves EV bioactivity than prolonged ultracentrifugation; however, membrane fouling and the co-retention of similarly sized contaminants may affect purity (Buschmann et al., 2021). Immunoaffinity capture provides high specificity by selectively enriching EV subpopulations through antibodies or aptamers targeting surface markers such as CD9, CD63, and CD81, thereby markedly improving EV purity, although the yield is generally lower and the method depends on the availability of well-characterized surface markers (Ragni, 2025). In contrast, precipitation methods typically achieve high recovery but frequently co-isolate abundant soluble proteins, lipoproteins, and polymer residues, resulting in relatively poor purity and potential interference with downstream functional analyses (Buschmann et al., 2021). Therefore, selection of an appropriate isolation strategy should consider not only EV recovery but also downstream quality requirements, including purity, biological activity, reproducibility, and intended biomedical applications. Given the difficulty of achieving all of the quality of EVs with a single method, combination strategies employing two or more methods are commonly adopted in practice, accompanied by multi-technique verification.

**TABLE 1:** Comparison of quality control among different EV isolation methods.

Isolation method	Recovery	Purity	Structural integrity	Biological activity	Major contaminants	Batch reproducibility	References
Ultracentrifugation	High	Moderate	Moderate	Moderate	Soluble proteins, protein aggregates	Moderate	(Gardiner et al., 2016)
Precipitation	High	Low	Moderate	Moderate-low	Soluble proteins, lipoproteins, polymer residues	High	(Buschmann et al., 2021)
Ultrafiltration	High	Moderate	High	High	Similarly sized particles, membrane-retained impurities	High	(Buschmann et al., 2021)
Immunoaffinity capture	Low	Very high	High	High	Minimal non-specific contaminants	Moderate	(Ragni, 2025)
SEC	Moderate	High	High	High	Minimal soluble proteins and lipoproteins	High	(Böing et al., 2014)

### 3.3 Engineered extracellular vesicles

Although native EVs possess inherent bioactivity, limitations such as insufficient targeting, limited therapeutic payload, and short *in vivo* half-life, have prompted a shift from passive utilization to active design. Engineered EVs refer to vesicles whose surface molecules and internal cargos have been directionally modified through chemical, biological, or physical techniques to enhance their targeting, stability, drug-loading efficiency, and therapeutic activity (Figure 2) (Zhang M. et al., 2023). Engineering strategies fall into two major categories, surface engineering and cargo engineering (Table 2).

**FIGURE 2:**
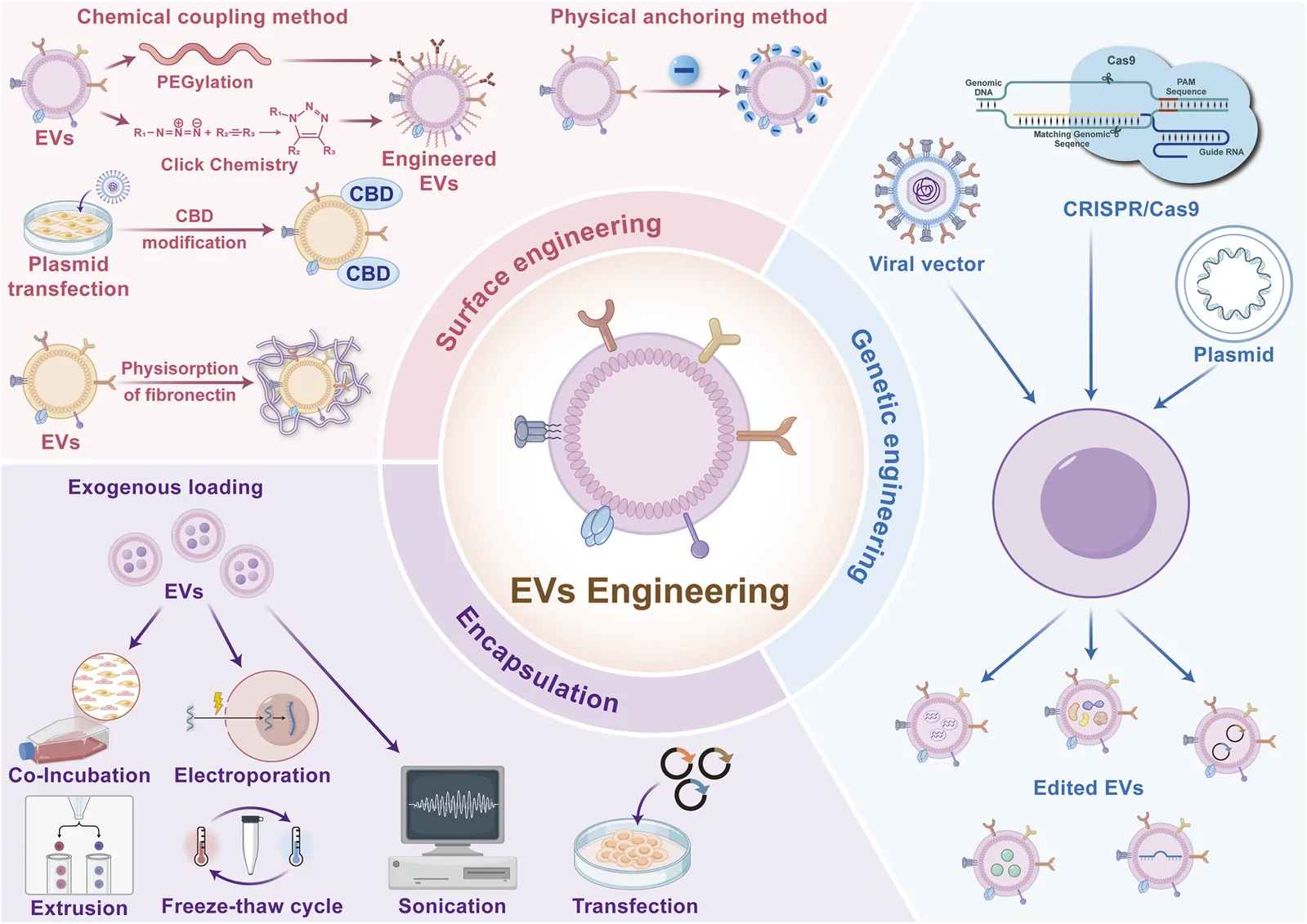
Overview of current EV engineering strategies for wound repair. EV engineering strategies are broadly categorized into surface engineering, genetic engineering, and encapsulation. Surface engineering modifies the EV membrane through chemical conjugation or physical anchoring to improve targeting, circulation stability, or tissue retention. Genetic engineering modifies donor cells using plasmids, viral vectors, or clustered regularly interspaced short palindromic repeats/CRISPR-associated protein 9 (CRISPR/Cas9) technology, enabling the production of EVs enriched with specific proteins or nucleic acids. Encapsulation introduces therapeutic molecules into EVs through exogenous approaches, including co-incubation, electroporation, extrusion, freeze–thaw cycling, sonication, and transfection. PEG, polyethylene glycol; CBD, collagen-binding domain; EVs, extracellular vesicles.

**TABLE 2:** Representative studies of engineered EVs for wound healing.

Study type	Wound model	EV type	Engineering approach	Loading material	Purpose of engineering	Hydrogel/delivery approach	Targeted mechanism	Main treatment effects
*In vitro* + *in vivo* (mouse)	STZ-induced diabetic mouse full-thickness skin wound	Endothelial progenitor cell-derived biomimetic nanovesicles	Lipid insertion	cRGD peptide	Enhance endothelial cell targeting and local delivery of EVs	Fe^3+^@PA-modified ADM/GelMA dual-network hydrogel	Modulation of oxidative stress and inflammation	Accelerated diabetic wound closure; increased neovascularization; enhanced collagen deposition and skin regeneration (Liu S. et al., 2024)
*In vitro* + *in vivo* (mouse)	db/db diabetic mouse skin wound	HEK293T-derived EVs	Transfection (virus)	Serpin	Restore deficient Serpin proteins in diabetic EVs and enhance their wound-repair activity	Local EV administration; no hydrogel	Restoration of Serpin deficiency; regulation of protease activity	Accelerated wound closure, enhanced re-epithelialization, and improved tissue repair (Park et al., 2022)
*In vitro* + *in vivo* (mouse)	db/db diabetic mouse full-thickness skin wound	MSC-derived EVs	Transfection (plasmid)	HOTAIR	Enrich EVs with HOTAIR to enhance pro-angiogenic activity	Local EV administration; no hydrogel	Upregulation of VEGF expression and promotion of angiogenic signaling	Enhanced angiogenesis, accelerated wound closure (Born et al., 2022)
*In vitro* + *in vivo* (mouse)	STZ-induced diabetic mouse full-thickness skin wound	ADSC-derived EVs	Transfection (virus)	BCL-2	Enhance angiogenesis and tissue repair	epsilon-poly-lysine and hyaluronic acid antimicrobial hydrogel	Enrichment of PI3K-Akt and AGE-RAGE signaling pathways; enhanced growth factor activity and angiogenesis; suppression of inflammatory cytokines (TNF-α and IL-6)	Accelerated diabetic wound healing; enhanced angiogenesis and cell proliferation; reduced inflammation (Ju et al., 2025)
*In vitro* + *in vivo* (rat)	Rat skin burn wound	MSC-derived EVs	Co-incubation	Iron oxide nanoparticle	Enable magnetic targeting and increase EV accumulation at the wound site	Systemic injection; no hydrogel	Magnetic targeting enhances EV accumulation at the wound site; upregulation of VEGFA and CXCL12 expression	Accelerated wound closure, enhanced angiogenesis and collagen deposition, increased re-epithelialization, and reduced scar formation (Li X. et al., 2020)
*In vitro* + *in vivo* (mouse)	db/db diabetic mouse skin wound	Macrophage-derived EVs	Exofect	MiR-425-5p	Enrich EVs with MiR-425-5p	Local EV administration; no hydrogel	miR-425-5p-mediated upregulation of adiponectin in dermal fibroblasts, promoting macrophage-fibroblast intercellular communication and keratinocyte proliferation	Accelerated diabetic wound closure and improved re-epithelialization (Park et al., 2024)
*In vitro* + *in vivo* (mouse)	Excisional wound and diabetic wound	HEK293T-derived EVs	Transfection (plasmid)	HIF-1α	Deliver stable HIF-1α to enhance hypoxia-responsive signaling and angiogenesis	Collagen sponge scaffold	Activation of HIF-1α signaling and upregulation of hypoxia-responsive angiogenic genes	Enhanced angiogenesis, accelerated wound closure (Jeon et al., 2025)
*In vitro* + *in vivo* (mouse)	Excisional wound and diabetic wound	HEK293T-derived EVs	Surface engineering Transfection (plasmid)	CBD	Enhance collagen binding, wound-site retention	Collagen sponge scaffold	CBD-mediated collagen targeting enhances EV retention and sustained local delivery	Enhanced angiogenesis, accelerated wound closure (Jeon et al., 2025)
*In vitro*	*In vitro* endothelial-cell model	MSC-derived EVs	mRNA Transfection	VEGF mRNA	Enrich EVs with VEGF mRNA	EV treatment *in vitro*; no hydrogel	VEGF-mediated activation of pro-angiogenic and anti-apoptotic signaling in endothelial cells	Enhanced endothelial cell survival, increased angiogenic activity (Zhang et al., 2026b)
*In vitro* + *in vivo* (mouse)	Mouse full-layer skin defect wound	BMSC-derived ApoEVs	Co-incubation	sPLA2-X	Modify ApoEV lipid metabolism to enhance macrophage uptak	Local EV administration; no hydrogel	sPLA_2_-X-mediated phospholipid hydrolysis increases resolvin D5 (RvD5) production, enhances macrophage uptake of ApoEVs, and suppresses TNF-α expression	Enhanced macrophage-mediated tissue repair (Qin et al., 2025)
*In vitro* + *in vivo* (mouse)	STZ-induced diabetic mouse full-thickness skin wound	ADSC-derived EVs	Transfection (virus)	Tβ4	Enrich ADSC-EVs with Tβ4 to alleviate cellular senescence	Separable GelMA/PEGDA microneedle patch with HA backing layer	Activation of the PTEN/PI3K/AKT signaling pathway to alleviate cellular senescence	Reversed cellular senescence, accelerated diabetic wound closure, enhanced angiogenesis and collagen deposition (Ding Y. et al., 2025)

Surface engineering introduces functional molecules onto the EV outer membrane to confer new capabilities such as targeted recognition, immune evasion, or tissue anchoring (Raghav and Jeong, 2021; Liao et al., 2024; Jang et al., 2025; Hong et al., 2026). Three main strategies are employed. Chemical conjugation uses covalent bonds to attach functional molecules to amino or sulfhydryl groups of EV membrane proteins, as in click chemistry, PEGylation, or direct peptide conjugation, offering high modification stability to address the poor targeting and rapid clearance of native EVs (Morishita et al., 2024). Physical anchoring integrates functional modules into the EV membrane via lipid insertion or electrostatic adsorption, for example, the insertion of cRGD peptides onto the surface of endothelial progenitor cell-derived nanovesicles via lipid anchors to achieve active endothelial cell (EC) targeting and promote angiogenesis in diabetic wounds (Németh et al., 2022; Liu S. et al., 2024). Genetic strategies involve plasmid transfection or lentiviral transduction of source cells to display genes encoding targeting peptides or functional proteins on the EV surface, exemplified by the introduction of a collagen-binding domain (CBD) gene into HEK293T cells to secrete EVs surface-displaying CBD, which substantially enhances EV retention and local enrichment in collagen-rich wounds (Jeon et al., 2025). In the realm of wound repair, surface-engineered EVs have undergone preliminary validation: fibronectin-adsorbed adipose-derived stem cells (ADSCs)-derived EVs significantly enhanced osteoblast spreading and upregulated alkaline phosphatase, type I collagen, and osteocalcin gene expression, while iron oxide-labeled human mesenchymal stem cell (MSC)-derived EVs under magnetic navigation guidance markedly improved enrichment at skin injury sites, promoting EC proliferation, migration, and angiogenesis while reducing scar formation (Li X. et al., 2020; Chen et al., 2021).

Cargo engineering aims to increase the loading capacity and functional activity of therapeutic molecules inside EVs. Loading strategies comprise physical methods (electroporation, sonication, freeze-thaw, extrusion), chemical/biological methods (co-incubation, chemical transfection reagents, lentiviral transduction, CRISPR/Cas9, and endogenous overexpression), in which source cells are modified to upregulate specific therapeutic molecules that are subsequently packaged into EVs (Jia et al., 2021; Born et al., 2022). Endogenous overexpression is the most widely applied strategy in wound repair because it fully leverages the cell’s natural loading machinery with minimal structural damage to EVs. Representative work includes the introduction of a sequence encoding constitutively active hypoxia-inducible factor-1 alpha (HIF-1α) into HEK293T cells for functional loading and sustained delivery of HIF-1α within EVs (Jeon et al., 2025); macrophage-derived EVs loaded with miR-425-5p via Exofect transfection, which accelerate inflammation resolution and epithelial regeneration in diabetic wounds through regulation of adiponectin expression; vascular endothelial growth factor (VEGF) mRNA-engineered MSC-EVs with markedly enhanced EC anti-apoptotic capacity; and thymosin β4 (Tβ4)-engineered ADSC-EVs delivered via detachable microneedle patches to rescue cellular senescence in diabetic wounds (Park et al., 2024; Ding Y. et al., 2025; Zhang et al., 2026b). Bcl-2-engineered ADSC-EVs have also been reported to promote angiogenesis and tissue repair (Ju et al., 2025). Beyond nucleic acid and protein loading, sPLA2-X-treated stem cell-derived apoptotic vesicles (ApoEVs) significantly increased the production of anti-inflammatory lipid mediators, promoted macrophage uptake, and suppressed TNF-α expression, thereby accelerating skin wound healing, highlighting lipid cargo engineering as an important expansion direction for EV cargo modification (Qin et al., 2025).

In summary, surface engineering confers targeting and anchoring capacity while cargo engineering enhances therapeutic molecular payload; these approaches address different limitations of native EVs. (Hu et al., 2024; Qin et al., 2024). Balancing functional enhancement with preservation of intrinsic EV properties remains an important challenge.

## 4 Classifications and design principles of hydrogels

The 3D hydrophilic network of hydrogels not only provides a physical protective reservoir for EVs but also fundamentally determines the release behavior and wound-adaptation capacity of the composite system through its intrinsic properties, including source, chemical composition, crosslinking mode, and environmental responsiveness (Figure 3). This chapter discusses hydrogel design principles for EV delivery at three progressive levels: material classification, crosslinking strategies, and stimuli-responsive design.

**FIGURE 3:**
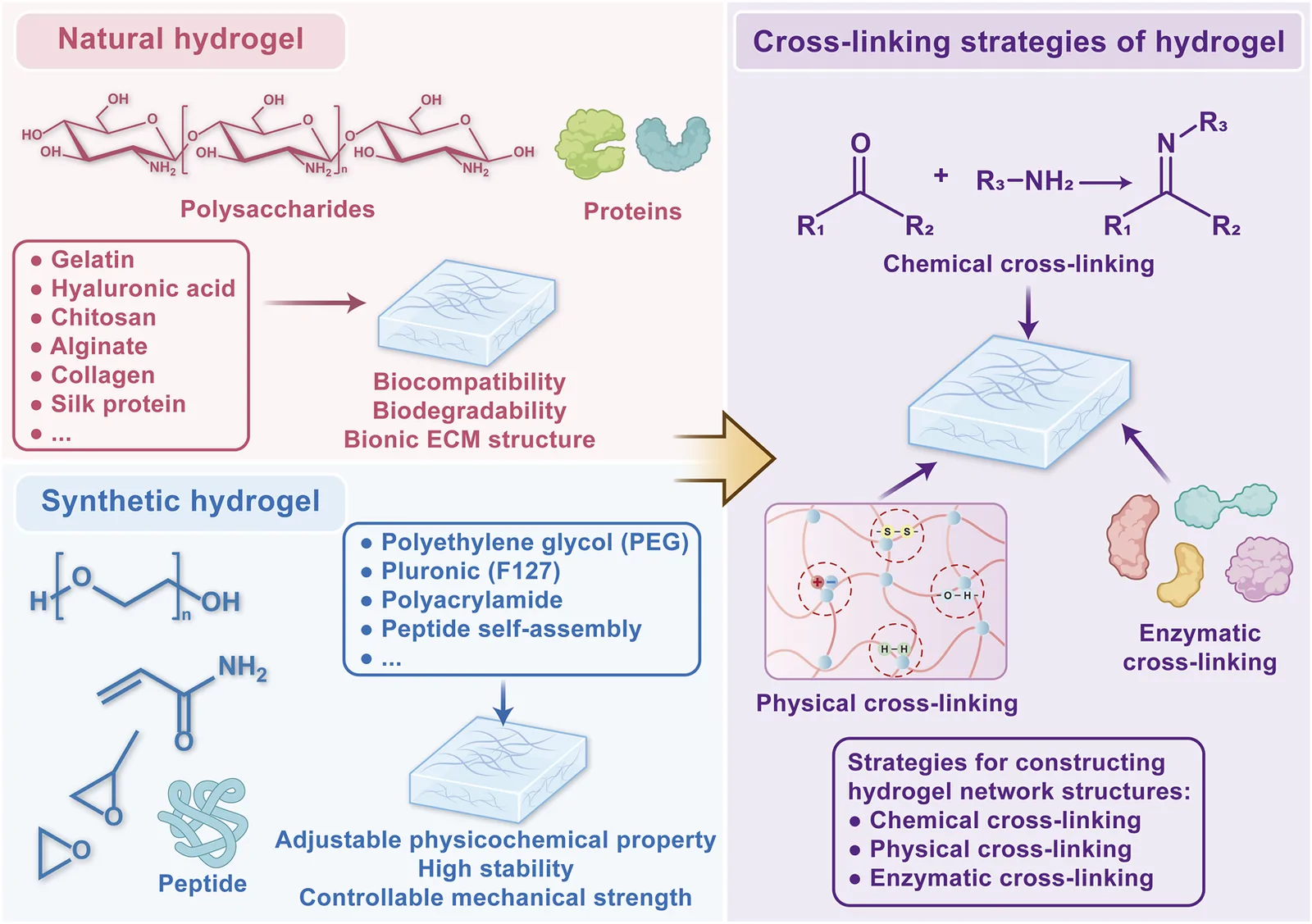
Classification and crosslinking strategies of biomaterial hydrogels for EV delivery. Hydrogels are broadly classified as natural or synthetic according to their material origin. Natural hydrogels generally exhibit favorable biocompatibility, biodegradability, and extracellular matrix (ECM)-mimicking properties, whereas synthetic hydrogels provide greater tunability of physicochemical and mechanical properties. Hydrogel networks can be formed through chemical, physical, or enzymatic crosslinking, which influence network stability, degradation, and EV retention and release. ECM, extracellular matrix; PEG, polyethylene glycol; EVs, extracellular vesicles.

### 4.1 Classifications of hydrogels

Based on raw material origin, hydrogels can be divided into natural and synthetic categories. Natural hydrogels are derived from biological macromolecules, primarily including polysaccharides (hyaluronic acid, chitosan, sodium alginate), whose molecular chains are rich in hydroxyl, carboxyl, and amino groups amenable to chemical modification and crosslinking; proteins (gelatin, silk fibroin, collagen), which contain cell adhesion sequences such as arginine-glycine-aspartic acid (RGD) that mimic the biochemical signals of native ECM; and gelatin methacryloyl (GelMA), which combines the bioactivity of natural origin with the controllability of photo-crosslinking and is currently one of the most widely used natural-derived hydrogels (Tang et al., 2023; Deng et al., 2025; Galbiati et al., 2025). Natural hydrogels generally exhibit favorable biocompatibility and inherent bioactivity, but are limited by batch-to-batch compositional variation, moderate mechanical strength, and difficulty in precisely tuning degradation rates. Synthetic hydrogels are constructed from artificial polymers, with representative materials including polyethylene glycol (PEG), poly (N-acryloyl glycinamide) (PNAGA), poly-γ-glutamic acid (γ-PGA), and polyurethanes (Liu W. et al., 2025). Their main characteristics include tunable physicochemical properties, relatively high batch-to-batch consistency, and adjustable mechanical properties. (Li Q. et al., 2020). Their drawback lies in the lack of intrinsic cell adhesion sites and enzyme-degradable sequences in most synthetic polymers, typically requiring incorporation of degradable crosslinkers to impart bioactivity.

For wound repair and EV delivery, hydrogels must integrate biocompatibility, aligned degradation kinetics, adequate hydration, skin-matching mechanics, and modifiable chemistries for EV retention and stimuli-responsive release. Composite hydrogels can combine the bioactivity of natural materials with the tunable physicochemical properties of synthetic polymers and therefore represent a useful design strategy for EV delivery.

### 4.2 Crosslinking strategies for hydrogel network formation

A stable 3D network constitutes the structural basis for long-term EV encapsulation and controlled release. Based on bonding mechanisms, hydrogel crosslinking strategies can be classified into physical, chemical, and enzymatic crosslinking, each differing fundamentally in reaction conditions, network stability, and impact on EV activity.

Chemical crosslinking constructs permanent 3D networks through stable covalent bonds between polymer chains, offering structural stability and high mechanical strength. Crosslinking density can be adjusted to regulate pore size, mechanical properties, degradation, and EV release. Representative approaches include photopolymerization, Schiff-base reactions, hydrazone formation, and natural crosslinker-mediated reactions. For example, methacryloyl-modified polymers such as GelMA are polymerized under ultraviolet or visible light irradiation catalyzed by a photoinitiator, with rapid reaction under mild conditions permitting simultaneous EV encapsulation and *in situ* gelation (Weng et al., 2025). Another strategy relies on Schiff base reactions, in which aldehyde groups of oxidized polysaccharides condense with amino-containing polymers to form dynamic imine bonds that proceed spontaneously under physiological conditions, with the reversibility of imine bonds conferring self-healing potential (Yue et al., 2024). Hydrazone bond crosslinking is formed via condensation of aldehydes with hydrazide groups, where the dynamic covalent nature allows local segment reorganization while maintaining overall network stability, suitable for constructing injectable granular hydrogels (Muir et al., 2022). Natural crosslinker-mediated crosslinking involves reagents such as genipin, which reacts with primary amines on polymer chains to form blue-colored covalent networks with controllable crosslinking density and markedly lower cytotoxicity than conventional reagents such as glutaraldehyde (Shu et al., 2023). A principal advantage of chemical crosslinking lies in tunable network architecture and strong controlled-release capability, making it suitable for chronic wounds requiring long-term sustained drug delivery. However, free radicals or reactive intermediates generated during crosslinking may compromise the membrane proteins and nucleic acid integrity of encapsulated EVs, necessitating a careful balance between crosslinking efficiency and EV activity preservation.

Physical crosslinking relies on non-covalent interactions including hydrogen bonding, electrostatic interactions, ionic complexation, hydrophobic association, and chain entanglement to construct reversible networks without chemical crosslinkers or initiators, offering mild preparation conditions particularly conducive to preserving EV bioactivity. Ionic crosslinking is the most commonly employed physical crosslinking mode, with the classic egg-box structure formed through electrostatic coordination between carboxyl groups of sodium alginate and divalent cations such as Ca^2+^, enabling rapid gelation under mild conditions (Gil-Cabrerizo et al., 2022). Temperature-induced gelation exploits the lower critical solution temperature behavior of polymer chains, with spontaneous sol-to-gel transition at body temperature, as exemplified by sodium alginate derivatives forming *in situ* reservoirs through thermally induced entanglement of double-helical junction zones for sustained local EV delivery (Wang B. et al., 2026). The main advantages of physical crosslinking are mild processing, absence of toxic residues, and preservation of the native conformation and bioactivity of EVs; its limitations include generally lower mechanical strength compared to chemical crosslinking systems and *in vivo* stability dependent on local environmental factors, potentially leading to batch-to-batch variation in EV release behavior. In addition, enzymatic crosslinking utilizes enzymes such as transglutaminase and horseradish peroxidase to catalyze the reaction of specific functional groups and form covalent hydrogel networks. This crosslinking method is characterized by mild reaction conditions and favorable biocompatibility (Li J. et al., 2024).

Overall, chemical crosslinking excels in structural controllability, physical crosslinking prioritizes EV activity preservation, and enzymatic crosslinking offers a unique balance between mildness and stability. Most current studies focus on optimizing a single modality, and head-to-head comparison of different strategies under equivalent conditions remains unexplored (Garcia-Aponte et al., 2025). Integrating multiple strategies is emerging as an important direction for simultaneously ensuring network stability and EV activity.

### 4.3 Hydrogel design principles for EV delivery in wound healing

#### 4.3.1 Universal design principles

Hydrogel functions not only as a carrier for EVs but as a bioactive platform capable of regulating the wound microenvironment. Regardless of wound type, several universal design principles should be considered. First, hydrogels should maintain a moist wound environment with appropriate water retention and vapor permeability, which facilitates keratinocyte migration, collagen deposition, and re-epithelialization while preventing secondary tissue dehydration. Second, hydrogel should mimic the native ECM by providing a highly hydrated three-dimensional porous network that supports cell adhesion, nutrient diffusion, and tissue remodeling. Third, hydrogels should protect EVs from rapid enzymatic degradation and systemic clearance while preserving their structural integrity and biological activity. Furthermore, appropriate mechanical properties, including injectability, tissue adhesiveness, flexibility, self-healing capability, together with favorable biocompatibility and biodegradable behavior, are essential to ensure intimate wound contact and gradual replacement of the scaffold by regenerated tissue. These shared characteristics constitute the fundamental design principles of EV-loaded hydrogels (Yan et al., 2023; Gao et al., 2026).

#### 4.3.2 Wound microenvironment-guided hydrogel design

Although universal characteristics are indispensable, wound-specific pathological microenvironments ultimately determine the rational design of EV-loaded hydrogels.

Acute wounds generally undergo an orderly healing process with transient inflammation and relatively preserved regenerative capacity. Therefore, hydrogel design mainly aims to maintain moisture, provide ECM-like structural support, and improve the local retention of EVs. Sustained EV release promotes fibroblast proliferation, angiogenesis, collagen deposition, and rapid re-epithelialization without requiring sophisticated stimuli-responsive functions (Cañedo-Dorantes and Cañedo-Ayala, 2019).

Unlike acute wounds, diabetic wounds are characterized by hyperglycemia, persistent oxidative stress, chronic inflammation, bacterial biofilm formation, tissue hypoxia, abnormal pH and elevated MMP activity (Zhang S. et al., 2023; Zheng et al., 2025), all of which compromise EV stability and markedly reduce therapeutic efficacy. Therefore, hydrogel design has shifted from passive EV carriers toward microenvironment-responsive delivery platforms that regulate EV release according to the complex wound microenvironment (Table 3). Among endogenous-responsive systems, ROS-responsive hydrogels are the most extensively investigated because excessive ROS accumulation represents a hallmark of chronic diabetic wounds. For example, Pan et al. developed a diselenide-crosslinked PVA-CS@HA hydrogel containing ROS-sensitive linkages that released kiwifruit-derived EVs in response to elevated ROS while reducing oxidative damage, thereby promoting diabetic wound repair (Pan et al., 2026). Likewise, glucose-responsive hydrogels utilize phenylboronic acid chemistry or glucose oxidase-mediated reactions to achieve glucose-triggered EV release. Yang et al. reported a boronate ester-crosslinked hydrogel loaded with *Houttuynia Cordata Thunb*-derived EVs, which responded to hyperglycemia and accelerated diabetic wound healing through modulation of the NF-κB and YAP signaling pathways (Yang W. et al., 2025). In addition, enzyme-responsive hydrogels exploit the overexpression of MMPs in diabetic wounds by incorporating protease-cleavable peptide sequences into the hydrogel network. Such systems enable pathological enzyme-triggered degradation and controlled EV release. Meng et al. demonstrated that an MMP-responsive hydrogel achieved programmed release of M2 macrophage-derived EVs, facilitating macrophage polarization toward the reparative M2 phenotype and accelerating the transition from inflammation to tissue regeneration (Meng et al., 2025). Besides endogenous cues, several externally regulated stimuli-responsive hydrogels, including near-infrared light, ultrasound, and thermoresponsive systems, have recently been developed to improve spatiotemporally controlled cargo release (Wang B. et al., 2026). However, most of these platforms have thus far been evaluated using therapeutic drugs, metal ions, or bioactive molecules rather than EVs, and their application as EV delivery systems remains largely at the proof-of-concept stage. Future studies integrating these externally controlled platforms with EV therapy may provide greater flexibility for personalized wound management. More recently, rather than responding to a single pathological signal, dual-responsive and multi-responsive hydrogels may be explored as a strategy to address the complex microenvironment of refractory wounds (Zhang Y. et al., 2025; Zhao et al., 2026).

**TABLE 3:** Representative stimuli-responsive hydrogels for treatment of wound healing.

Response signals	Core materials	Cargo/system type	Quantitative response characteristics	Wound-relevant efficacy	Responsive mechanisms
ROS	PVA-CS@HA	EV-loaded hydrogel	95% release and 35% mass loss at 60 h under 0.01 M H_2_O_2_, versus 31.2% release and 7.9% mass loss in PBS; stimulus threshold/sensitivity and repeatability: NR	Promoted diabetic wound repair *in vivo*	Synthesizing and incorporating ROS-cleavable crosslinkers (Pan et al., 2026)
Glucose	rhCOL3/3-aminophenylboronic acid	EV-loaded hydrogel	Stimulus threshold/sensitivity, response time, and repeatability: NR	Accelerated diabetic wound healing *in vivo* through modulation of NF-κB and YAP signaling	A boronate ester-crosslinked hydrogel responded to hyperglycemia (Yang W. et al., 2025)
MMP-9	Oxidized dextran	EV-loaded hydrogel	Stimulus threshold/sensitivity, response time, and repeatability: NR	Promoted M2 macrophage polarization and diabetic wound repair *in vivo*	Cross-linking of oxidized dextran, a matrix metalloproteinase (MMP)-9-sensitive peptide (Meng et al., 2025)
Glucose and pH	HAP/OCS/PEG/Ag-E	EV-loaded hydrogel	Response evaluated at pH 5.5 and 33 mM glucose; structural response observed within 1 h at 37 °C; both conditions enhanced EV release; stimulus threshold/sensitivity and repeatability: NR	Accelerated diabetic wound healing with reduced inflammation and enhanced angiogenesis and collagen deposition	Hyaluronic acid-phenylboronic acid conjugate (HAP), enabling pH/glucose-responsive crosslinking via Schiff base (mainly pH-responsive) and phenylboronic ester bonds (both pH and glucose-responsive). Oxidized chondroitin sulfate (OCS) introduced aldehyde groups for additional Schiff base crosslinking, while its ortho-dihydroxy groups facilitated borate ester formation with HAP (Zhang Y. et al., 2025)
Glucose and oxidative stress	GPP@ZnBG	Non-EV cargo-loaded hydrogel (cargo zinc, calcium, and silicate ions)	Tested under 25 mM glucose and 100 μM H_2_O_2_; ion-release responses evaluated from 12 h to 14 days; stimulus threshold/sensitivity and repeatability: NR	Accelerated diabetic wound healing with improved neovascularization and enhanced collagen deposition	Upon exposure to pathological glucose/ROS levels, phenylboronate ester bonds cleave, triggering responsive degradation of the hydrogel matrix (Zhao et al., 2026)

NR, Not reported. The non-EV system is included only as a design reference for potential adaptation to EV delivery. Quantitative stimulus concentrations represent experimentally tested conditions and should not be interpreted as validated response thresholds unless explicitly established in the original study.

Burn wounds are characterized by extensive tissue necrosis, severe inflammatory responses, excessive exudation, and a high risk of microbial infection and hypertrophic scar formation (Wang et al., 2018). Therefore, EV-loaded hydrogels for burn treatment should possess strong exudate absorption, broad-spectrum antibacterial activity, prolonged EV retention, and anti-fibrotic capability. Recent hydrogel designs for melanoma post-resection wounds have integrated local drug delivery to promote wound healing and potentially reduce postoperative tumor recurrence risk (Chen S. et al., 2024; Liu X. et al., 2024). Although these multifunctional hydrogels have not yet been engineered as EV delivery systems, their design concepts provide a framework for the future development of EV-loaded hydrogels for the management of post-resection skin tumor wounds. Chronic refractory wounds, such as pressure ulcers, venous leg ulcers, and radiation-induced wounds, share common pathological features including prolonged inflammation, impaired angiogenesis, and defective ECM remodeling (Pastar et al., 2023). Consequently, hydrogel design should be tailored to the dominant pathological barriers of each wound.

Overall, hydrogel design has evolved from conventional biomaterial scaffolds toward microenvironment-guided therapeutic platforms. Rather than focusing solely on the physicochemical properties of biomaterials, current strategies increasingly integrate wound-specific pathological characteristics including oxidative stress, glucose dysregulation, protease imbalance, infection, hypoxia, and chronic inflammation. This pathology-oriented approach provides a rational framework for the design of EV-loaded hydrogels tailored to different wound conditions. Nevertheless, quantitative characterization of stimuli-responsive hydrogels remains limited, particularly regarding stimulus thresholds, sensitivity, specificity, response time, reproducibility and quantitative efficacy under wound-relevant conditions, highlighting the need for more systematic evaluation of these parameters in future studies.

## 5 Strategies for EV loading by hydrogels

To accommodate the structural characteristics and therapeutic requirements of different wound types and achieve sustained and effective retention of EVs at the wound site, extensive research has been devoted to identifying the optimal composite strategy. Based on the timing of EV introduction relative to hydrogel crosslinking, current strategies can be classified into four types: combining EVs after crosslinking, crosslinking after combining EVs, *in situ* crosslinking, and three-dimensional (3D) bioprinting (Figure 4).

**FIGURE 4:**
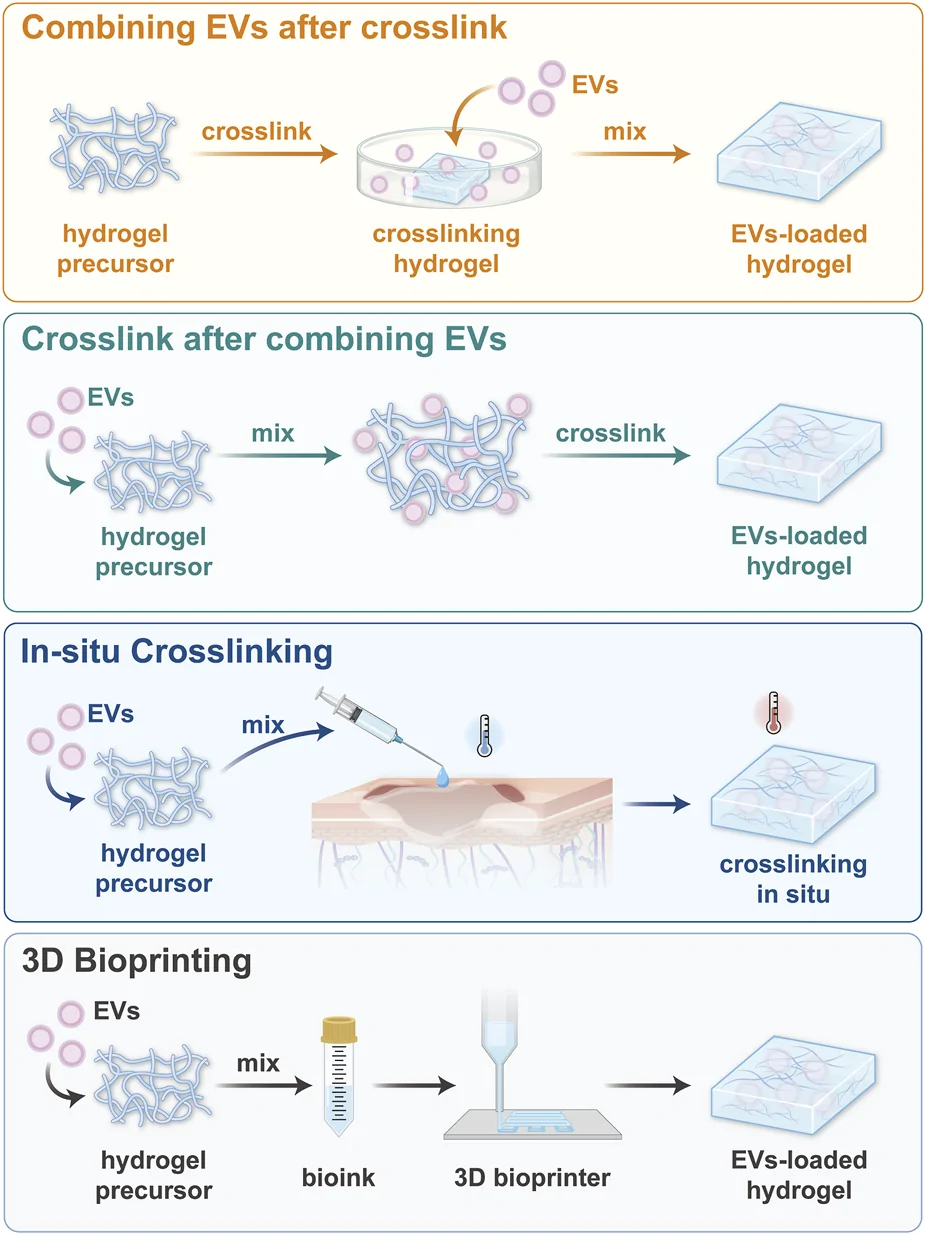
Strategies for integrating EVs with hydrogels. According to the timing and method of EV incorporation relative to hydrogel network formation, EV-hydrogel integration can be broadly categorized into four strategies: (1) incorporation of EVs into preformed hydrogels, (2) crosslinking after mixing EVs with hydrogel precursors, (3) *in situ* crosslinking of EV-containing hydrogel precursors at the treatment site, and (4) three-dimensional (3D) bioprinting of EV-containing bioinks. EVs, extracellular vesicles; 3D, three-dimensional.

### 5.1 Combining EVs after crosslink

In combining EVs after crosslinking or so-called post-loading, the hydrogel network is first formed via crosslinking, after which the dehydrated hydrogel is immersed in an EV-containing solution, allowing EVs to be passively adsorbed into the network through the swelling process. This approach is the simplest in procedure, and because EVs do not directly contact the crosslinking reaction, it is particularly suitable for EV systems sensitive to chemical crosslinking conditions such as free radicals or reactive intermediates. However, loading efficiency remains constrained by the pore-size barrier: pores must exceed EV dimensions to permit entry, yet larger pores compromise encapsulation and promote burst release. Moreover, EVs are retained solely through weak physical confinement and non-covalent interactions. Therefore, this method is particularly suitable for EV systems that are sensitive to crosslinking conditions and require avoidance of chemical or physical stimulation during hydrogel preparation.

### 5.2 Crosslink after combining EVs

In this strategy, EVs are uniformly dispersed in the hydrogel precursor solution and subsequently entrapped within the forming network through physical (e.g., thermal) or chemical (e.g., photo-initiated, ionic) crosslinking. Relative to post-loading, this method achieves more homogeneous EV distribution and stronger incorporation. Crosslinking conditions, particularly temperature and gelation rate, must nevertheless be carefully controlled to preserve EV structural integrity and bioactivity. Photo-crosslinked GelMA systems exemplify this balance, enabling efficient EV encapsulation under relatively mild conditions, while methacrylic anhydride-modified silk fibroin (SerMA) combined with royal jelly-derived EVs has adopted a similar approach with high loading efficiency and functional retention (Tan et al., 2023; Deng et al., 2025).

### 5.3 *In-situ* crosslinking

The *in-situ* crosslinking strategy delivers a mixture of EVs and liquid hydrogel precursors directly to the wound bed, where gelation is triggered by the local environment. Thermosensitive hydrogels are representative examples of this approach. They remain flowable at room temperature and undergo rapid gelation upon exposure to body temperature at the wound site, thereby entrapping EVs within a conformal depot (Wang B. et al., 2026). This strategy achieves intimate contact with irregular wound topography and prolongs local EV retention.

### 5.4 3D bioprinting

3D bioprinting employs additive manufacturing to construct scaffolds with defined spatial architecture and biological functionality through layer-by-layer deposition of bioinks, formulations typically comprising hydrogels, cells, ECM components, and growth factors that preserve cell viability and recapitulate the native tissue microenvironment. In tissue repair, this technology offers controlled fabrication of scaffold morphology, pore architecture, mechanical properties, and the spatial distribution of bioactive cues, thereby directing cell behavior and tissue regeneration (Zheng et al., 2024). Applied to hydrogel-EV composite systems, co-printing EVs with hydrogel bioinks enables programmed spatial distribution and controlled release, further enhancing therapeutic efficacy in complex wound repair (Jiao et al., 2023). For instance, a 3D-printed sodium alginate/hydroxyapatite (SA/HAP) hydrogel loaded with microalgae-derived EVs enhanced fibroblast viability and induced pro-angiogenic gene expression in MSCs, illustrating the benefits of hierarchical structural design (De Cesare et al., 2026).

In summary, the four strategies involve distinct trade-offs between operational convenience and EV retention controllability. Selection should be based on comprehensive consideration of wound characteristics, EV chemical sensitivity, and required release duration. Combining the complementary strengths of different strategies in future work may facilitate the development of wound-adapted EV delivery strategies.

### 5.5 EV loading, release kinetics and bioactivity from hydrogel systems

Beyond the mode of EV incorporation, quantitative characterization of EV loading and release is important for defining the effective dose and temporal availability of EVs within the wound microenvironment (Table 4). However, reporting remains heterogeneous across hydrogel-EV studies. Many studies report the initial EV amount or concentration rather than the actual loading or encapsulation efficiency, limiting direct comparison among delivery systems. Available studies nevertheless demonstrate markedly different release profiles. For example, an EV-loaded Q5 protein hydrogel exhibited an initial burst of 42% ± 1% at 1 h and 89% ± 5% at 2 h, followed by slower release (Britton et al., 2024), whereas genipin-crosslinked and recombinant collagen III hydrogels released >85% of EVs within 72 h and approximately 80% over 10 days, respectively (Li et al., 2021; Xu et al., 2022). Release duration can also be regulated by hydrogel network properties. Degradable PEG hydrogels enabled tunable EV release over approximately 6–27 days (Kwak et al., 2022), while altering photocrosslinking conditions produced release periods of approximately 2–7 days in a sprayable hydrogel system (Liu B. et al., 2025).

**TABLE 4:** Quantitative loading and release characteristics of representative hydrogel-EV systems for skin wound healing.

Study	EV type	Hydrogel system	EV loading efficiency	Burst release	Cumulative release	Release kinetics	Post-release EV bioactivity	Wound- healing phases
(Shen et al., 2021)	BMSC-sEVs	Bilayer thiolated alginate/PEGDA (BSSPD)	NR	NR	∼80% at day 7 (lower layer); ∼80% at day 9 (upper layer)	Sequential physical-diffusion release; different swelling/degradation rates	NR	B-sEVs for early inflammation/proliferation; miR-29b-3p-sEVs for late proliferation/maturation
(Li et al., 2021)	hUCMSC-derived EVs	Genipin-crosslinked hydrogel	NR	NR	>85% after 72 h	Sustained release; hydrogel degradation increased after 72 h	NR	NR
(Kwak et al., 2022)	M2 macrophage-derived EVs	Hydrolytically degradable PEG hydrogels	NR	Half-release time 8 h	Generally 100% 1–2 days before complete degradation	Tunable release over ∼6–27 days; release profile strongly correlates with hydrogel degradation	Released M2-EVs remained morphologically intact and functionally active for macrophage polarization	Overall kinetics matched initial inflammatory phase
(Xu et al., 2022)	hUCMSC-derived EVs	TGase-crosslinked recombinant human collagen III hydrogel	NR	NR	∼80% at day 10	Sustained release from TGase-crosslinked rhCol III	NR	NR
(Britton et al., 2024)	EVs	Q5 protein hydrogel	NR	42% ± 1% at 1 h; 89% ± 5% at 2 h	Subsequent release ∼4% per day until 72 h	Biphasic; later linear phase R^2^ = 0.91	NR	NR
(Lyu et al., 2024)	MSC-derived EVs	PGLADMA core/GelMA shell microneedle patch	NR	NR	25.24% ± 0.66% at day 14; 31.19% ± 0.27% at day 21	Ritger–Peppas: Q(t) = 3.93t^0.691; n = 0.691; anomalous diffusion/erosion transport	EVs remained structurally intact after 21-day release	Prolonged EV release aligned with proliferative phase
(Zhu et al., 2024)	3D-cultured ADMSC-derived EVs	Hyaluronan hydrogel	NR	NR	>65.1% at 48 h	Sustained release; mathematical model NR	NR	NR
(Liu B. et al., 2025)	hUCMSC-derived EVs	Photocrosslinking methacrylated decellularized dermal matrix	NR	NR	Exo@AMCN300: 74.67% ± 4.72% at 120 h and 87.67% ± 2.08% at 168 h	NR	NR	NR
(Wang et al., 2025)	Hypoxia-preconditioned ADSC EVs	HB-PEG/HA-SH *in situ* hydrogel	NR	NR	>75% by day 15	NR	Functional activity supported by uptake, scratch and tube-formation assays	NR
(Li S. et al., 2026)	Camel milk EVs	Thermosensitive hydrogel	NR	43.2% ± 3.1% at 8 h	81.7% ± 4.2% at 48 h	Higuchi R^2^ = 0.974 (best fit); diffusion-controlled release	NR	NR

NR, Not reported. EV loading amount or input concentration was not interpreted as loading efficiency. Post-release EV bioactivity was recorded as NR when biological activity was not specifically evaluated using EVs after release from the hydrogel. Quantitative values were included only when explicitly reported in the original studies and were not estimated from graphical data.

The temporal pattern of EV release may also be relevant to the changing biological requirements of wound healing. A bilayer hydrogel was designed to sequentially deliver bone marrow mesenchymal stem cells (BMSC)-derived sEVs during the early inflammatory and proliferative stages and miR-29b-3p-enriched sEVs during later proliferative and maturation stages (Shen et al., 2021). Similarly, prolonged EV release from a core-shell microneedle system was designed to overlap with the proliferative phase (Lyu et al., 2024). However, direct quantitative correlations between release kinetics and phase-specific biological responses remain limited. Preservation of EV bioactivity after release is another important consideration. Although some studies have demonstrated retained EV integrity or wound-relevant biological activity after hydrogel delivery, many evaluate the complete hydrogel-EV construct rather than EVs recovered after release, making it difficult to distinguish post-release EV potency from the contribution of the hydrogel itself.

Overall, quantitative comparison remains constrained by differences in EV source, hydrogel composition, EV input dose, release medium, sampling schedule, and analytical methods. Future studies should distinguish initial EV input from actual loading or encapsulation efficiency and consistently report burst release, cumulative release at defined time points, release duration, kinetic parameters, and post-release EV integrity and bioactivity. Such standardized reporting would facilitate comparison among hydrogel-EV systems and support the rational design of release profiles for different wound conditions and healing stages.

## 6 Mechanisms of hydrogel-EV systems in promoting wound healing

The wound-repair effects of hydrogel-EV systems involve multiple interconnected biological processes, including inflammatory regulation, angiogenesis, ECM remodeling, and reparative cell proliferation and migration. In addition, the hydrogel matrix can contribute to local EV retention, release control, and regulation of the wound microenvironment. This section discusses these biological processes and the complementary contribution of the hydrogel matrix to wound repair.

### 6.1 Regulation of inflammation and immunity

A core pathological hallmark of chronic non-healing wounds is a persistently excessive inflammatory state characterized by M1 macrophage hyperactivation, elevated expression of pro-inflammatory cytokines such as TNF-α, IL-6, and IL-1β, and massive ROS accumulation, trapping the wound in a prolonged inflammatory phase without progression to the proliferative repair stage. EV-loaded hydrogel can break this vicious inflammatory cycle through multiple pathways (Figure 5).

**FIGURE 5:**
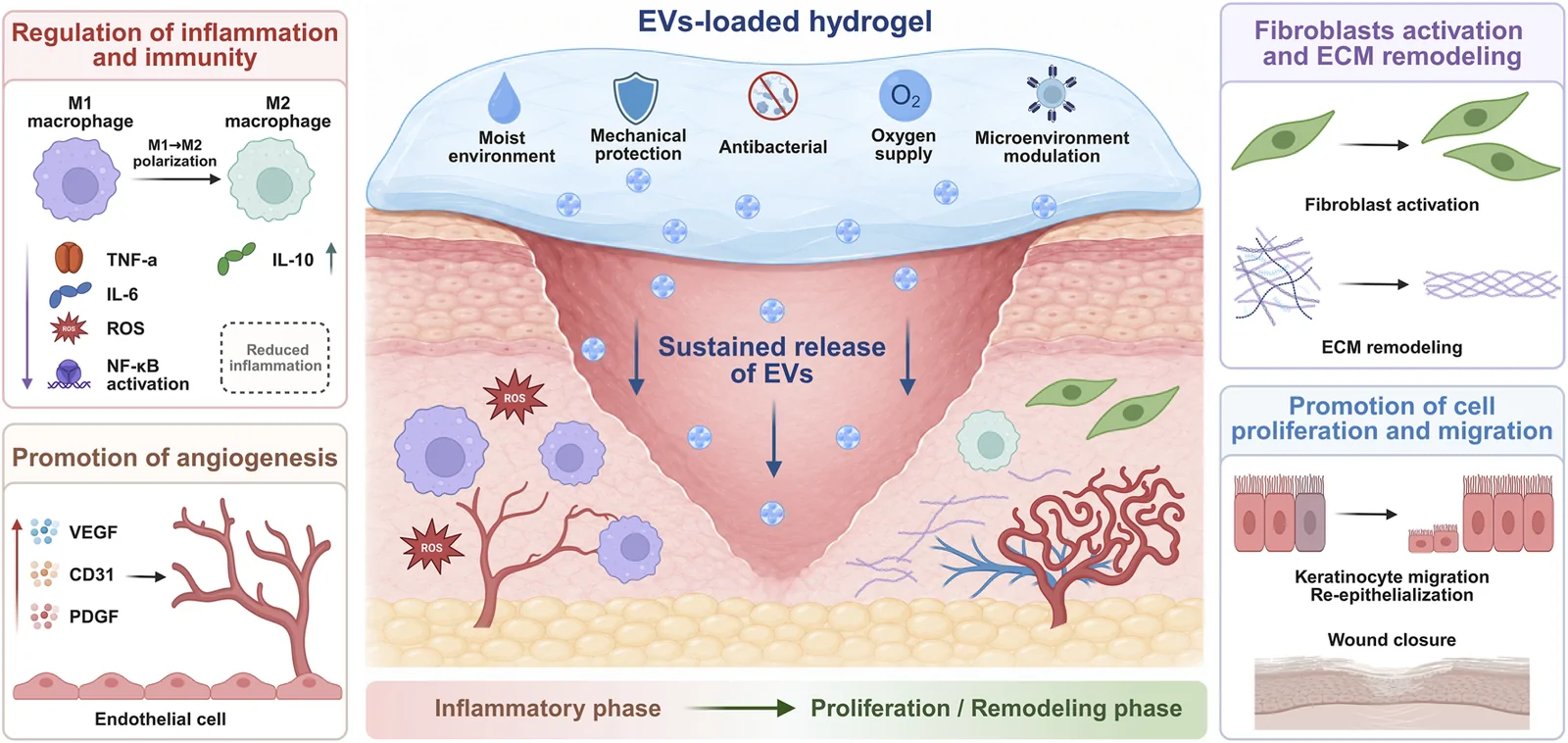
Biological mechanisms by which hydrogel-EV systems promote wound healing. Hydrogel-mediated EV delivery can regulate multiple interconnected processes involved in wound repair, including inflammatory modulation, angiogenesis, extracellular matrix (ECM) remodeling, and cell proliferation and migration. EV cargos can influence macrophage polarization and inflammatory cytokine production, promote endothelial-cell migration and neovascularization, regulate fibroblast activity and collagen deposition/remodeling, and enhance keratinocyte proliferation and re-epithelialization. EVs, extracellular vesicles; ECM, extracellular matrix; ROS, reactive oxygen species; VEGF, vascular endothelial growth factor; PDGF, platelet-derived growth factor.

The primary mechanism involves the regulation of macrophage phenotypic switching. Anti-inflammatory miRNAs and cytokines delivered by EVs can induce macrophage polarization from the pro-inflammatory M1 to the anti-inflammatory M2 phenotype, reducing pro-inflammatory cytokine expression while upregulating anti-inflammatory mediators such as IL-10 (Back et al., 2025). Deng et al. reported that Mo-EVs directly drove M2 polarization, and when encapsulated in GelMA hydrogel, significantly reduced wound area and increased epidermal thickness in a rat skin defect model (Deng et al., 2025). Zhang et al. constructed a hydrogel system loaded with *Lactobacillus* reuteri-derived EVs that promoted M2 polarization by reducing intracellular ROS levels and inhibiting NF-κB pathway activation, effectively alleviating the pro-inflammatory microenvironment of diabetic wounds (Zhang Y. S. et al., 2026).

Second, EVs can directly suppress pro-inflammatory cytokine expression. A composite hydrogel loaded with plant (Paris polyphylla)-derived EVs (PPEVs) significantly reduced wound IL-1β, IL-6, and TNF-α levels while scavenging excess ROS, achieving nearly 100% healing in full-thickness skin defect mice by day 14 (Tang et al., 2025). Apoptotic cell-derived EVs (ApoEVs) increased the production of anti-inflammatory lipid mediators through the sPLA_2_-X-mediated phospholipid hydrolysis pathway, promoted macrophage uptake, and markedly suppressed TNF-α expression, inhibiting the wound inflammatory microenvironment at the lipid signaling level (Qin et al., 2025).

In particular, EVs not only exert anti-inflammatory effects in chronic inflammation but also participate in the initiation of physiological inflammation during early wound healing. Wooil Choi et al., using Rab27A knockout mice, demonstrated that neutrophil-derived EVs carry effector molecules including cathelicidin antimicrobial peptide, neutrophil granule proteins, and complement C5, and that their absence impairs neutrophil recruitment to the wound site and delays pathogen clearance, thereby retarding wound healing (Choi et al., 2026). This finding indicates that EV-mediated immune regulation exhibits temporal biphasicity, promoting moderate physiological inflammation in the early phase to initiate the repair cascade while driving inflammation resolution in the later phase to enable progression into the proliferative stage.

### 6.2 Promotion of angiogenesis

The establishment of a functional vascular network represents a landmark event in the transition from the inflammatory to the proliferative phase of wound healing, delivering essential oxygen and nutrients for granulation tissue formation, re-epithelialization, and tissue remodeling. Hydrogel-EV systems drive angiogenesis cooperatively through multiple signaling pathways and constitute one of the principal mechanisms by which the composite system accelerates wound healing.

Mo-EVs have been shown to promote the proliferation, migration, and tube formation of HUVECs. Mechanistically, this effect is mediated by the significant upregulation of CD31, VEGF, and platelet-derived growth factor (PDGF) expression via activation of the ERK1/2 pathway (Deng et al., 2025). GelMA hydrogel loaded with keratinocyte-derived EVs promoted wound microvascular reconstruction through the PI3K/AKT pathway (Li Q. et al., 2025). Periosteum-derived EVs released from hydrogels similarly exhibited significant EC functional activation and *in vivo* angiogenic effects (Han et al., 2022).

In recent years, researchers have gained a more refined understanding of the pathological mechanisms underlying impaired angiogenesis in diabetic wounds. Lu et al. revealed a metabolic–signaling coupling regulatory axis: in the diabetic wound microenvironment, neutrophils excessively release neutrophil extracellular traps (NETs), which induce ferroptosis in ECs through inhibition of the PI3K/AKT signaling pathway, directly causing impaired angiogenesis. An MSC-EV-loaded hydrogel system delivered functional mitochondria to neutrophils, promoting mitochondrial fusion and restoring their oxidative phosphorylation function, thereby reducing NET release. With declining NET levels, endothelial PI3K/AKT pathway activity was restored and angiogenesis proceeded normally (Lu et al., 2024). This signaling chain of mitochondrial transfer, NET suppression, ferroptosis reversal, and angiogenic restoration provide a new perspective for understanding the pro-angiogenic mechanisms of hydrogel-EV systems.

Additionally, EV cargo miRNA-mediated translational regulation represents another important dimension. Fibroblast-derived EVs loaded in hydrogels were taken up by ECs, and their carried miR-24-3p targeted and inhibited VHL E3 ubiquitin ligase, leading to increased HIF-1α protein stability and subsequent transcriptional activation of VEGFA and VEGFR2, driving new blood vessel formation (Chen Y. et al., 2025). This miR-24-3p/VHL/HIF-1α/VEGF axis reveals a critical node of post-translational angiogenic regulation by EVs.

### 6.3 Promotion of fibroblast activation and extracellular matrix (ECM) remodeling

Fibroblasts are the core effector cells for ECM synthesis and remodeling. Hydrogel-EVs drive the deposition and ordered organization of ECM components such as collagen and fibronectin by activating fibroblast proliferation, migration, and secretory functions. MSC-derived nanovesicles produced by cell shearing were taken up by dermal fibroblasts and dose-dependently activated the MAPK signaling pathway, promoting fibroblast proliferation and migration while enhancing the secretion of growth factors such as VEGF and PDGF and ECM proteins (Neupane et al., 2023). EC-derived EVs (EC-EVs) activated fibroblast proliferation and ECM remodeling programs through upregulation of the transcription factor ETV1, and *in vivo* experiments confirmed that this mechanism increased wound bed collagen density and fibroblast numbers (Yuan et al., 2026). At the carrier level, ECM hydrogels, themselves composed of natural ECM components, promote collagen deposition through a dual mechanism of material biomimicry plus vesicle delivery when combined with ADSC-EVs (ECM@EV system) (Song et al., 2023). Genipin-crosslinked gelatin/β-cyclodextrin hydrogels loaded with platelet-rich plasma (PRP)-derived EVs promoted angiogenesis, collagen formation, and re-epithelialization by activating autophagy while inhibiting apoptosis of HUVECs and human skin fibroblasts (Shu et al., 2023). This parallel regulatory mode of autophagy activation coupled with apoptosis inhibition expands the molecular dimension of ECM reconstruction.

### 6.4 Promotion of cell proliferation and migration

After wound formation, local reparative cells are substantially depleted by injury and apoptosis. The proliferation and migration of fibroblasts and keratinocytes respectively drive granulation tissue filling and re-epithelialization and constitute the direct cytological basis for wound closure.

In terms of promoting re-epithelialization, hydrogel-encapsulated EVs in db/db diabetic mice significantly shortened healing time and accelerated re-epithelialization by prolonging EV retention at the wound site and enhancing bioavailability (Britton et al., 2024). A PFF/sEVs dual physically crosslinked hydrogel based on PNAGA/γ-PGA alleviated the inhibition of fibroblast proliferation and migration under high-glucose conditions, thereby maintaining reparative cell viability and function in a metabolically stressed microenvironment (Liu W. et al., 2025). MSC-derived EVs play similarly extensive roles at this level. Umbilical cord-mesenchymal stem cell (UCMSC)-derived EVs loaded in GelMA hydrogel significantly upregulated PCNA and CK19 expression in wound tissue of diabetic rats, promoting keratinocyte proliferation and epidermal regeneration (Tang et al., 2023). ADSC-EVs loaded in an ε-polylysine/hyaluronic acid dual-crosslinked antibacterial hydrogel not only promoted angiogenesis and alleviated inflammation but also enhanced the proliferative activity of reparative cells, comprehensively improving wound tissue architecture in diabetic mice (Ju et al., 2025).

### 6.5 Contributions of hydrogel matrices

Hydrogels can contribute to EV delivery not only by retaining and releasing EVs but also through their intrinsic physicochemical properties and effects on the local wound microenvironment. At the physical microenvironment level, the high-water-content moist environment provided by hydrogels prevents wound desiccation and crusting, while the 3D network offers mechanical support and contact guidance for reparative cell migration. At the chemical microenvironment level, oxygen-releasing hydrogels improve wound hypoxia through local oxygen supply, providing a metabolic basis for EV-induced angiogenesis and collagen synthesis (Saberian, 2026), while antibacterial hydrogels such as the ε-polylysine/hyaluronic acid system create favorable microenvironmental conditions for EV-mediated immunomodulation and pro-repair functions through infection control and bacterial load reduction (Ju et al., 2025). At days 9 and 12, the hydrogel incorporating ADSC-derived EVs promoted wound healing in diabetic mice. Of note, the hydrogel-only group also achieved partial reduction in wound size in diabetic mice (Ju et al., 2025). At the signal integration level, multifunctional hydrogels can actively sense and respond to wound pathological signals. Yang et al. designed an rhCOL3/APBA, glucose-responsive hydrogel loaded with *Houttuynia Cordata Thunb*-EVs that simultaneously regulated the NF-κB pathway (suppressing inflammation) and the YAP pathway (promoting proliferation and angiogenesis), concurrently modulating inflammatory and proliferative pathways (Yang W. et al., 2025). In this study, both free plant-derived EVs and hydrogel-only treatment contributed to wound healing in diabetic mice, while the EV-loaded hydrogel exhibited enhanced therapeutic performance for wound repair (Yang W. et al., 2025).

In summary, hydrogel-EV systems drive wound repair through a temporally coordinated cascade in which early inflammation resolution initiates the repair program, mid-stage angiogenesis restores microcirculation, and subsequent ECM remodeling coupled with cell proliferation and migration achieves wound closure. These biological processes are interconnected through signaling crosstalk, while the hydrogel matrix additionally contributes to EV retention, release, and local microenvironmental regulation (Yang et al., 2024; Zheng et al., 2024).

## 7 Applications of hydrogel-EV systems in wound healing

In recent years, numerous preclinical studies have evaluated hydrogel-EV systems in different skin wound models and reported beneficial wound-repair effects. The distinct pathological characteristics of different wound types impose differentiated requirements on EV source selection, hydrogel design, and release kinetics. This chapter reviews the application status and design logic of hydrogel-EV systems across several clinical scenarios: acute wounds, diabetic chronic wounds, burn wounds and other wounds, with reference to representative studies (Table 5).

**TABLE 5:** EVs complexed with hydrogels for wound healing.

Wound type	The source of EVs	Hydrogel	Model	EV dose	Wound closure	Vascularization	Collagen deposition	Molecular markers	Other major findings
Full-thickness skin defect	Mo-inspired macrophage	GelMA	Rats	NR	Day 14 significantly accelerated	NR	NR	NR	Enhanced proliferation, migration, tube formation, and angiogenic factor expression (CD31, VEGF, PDGF); promoted M2 macrophage polarization; reduced wound area and increased epidermal thickness; promoted angiogenesis through the activation of the ERK1/2 signaling pathway (Deng et al., 2025)
Full-thickness skin defect	Fibroblast	GelMA	Mice	9 × 10^9^ particles/mL	Day 14 wound area: 0.62 mm^2^ (GelMA-EVs) vs. 3.12 mm^2^ (GelMA) vs. 4.40 mm^2^ (Control)	NR	NR	NR	Accelerated wound healing, with faster closure, enhanced epithelialization, increased vascularization, and reduced fibrosis (Galbiati et al., 2025)
Full-thickness skin defect	Royal jelly	SerMA	Mice	25 μg/mL	Day 4: 54.5%; Day 7: 87.6%; Day 14: 96.8%	NR	NR	NR	Anti-inflammatory and antioxidant effects; promoted L929 cell proliferation and migration *in vitro* (Tan et al., 2023)
Full-thickness skin defect	hUCMSCs	CMCS/GP	Rats	6 μg/300 μL	Day 14: 98.9% ± 1.2% (Hydrogel + EVs); 86.9% ± 3.6% (Hydrogel); 74.8% ± 4.3% (Control)	NR	NR	NR	Improved wound closure, re-epithelialization rates, collagen deposition in the wound sites, TNF-α and IL-1β decreased (Li et al., 2021)
Full-thickness skin defect	Youthful Brain	GelMA	Rats	100 μg/mL	Day 14: ≈95%	NR	NR	NR	Promotes scarless healing in aged skin, encouraged collagen deposition, angiogenesis, epidermal and dermal regeneration, and folliculogenesis (Wu et al., 2025)
Full-thickness skin defect	*Lactobacillus* casei	Carboxymethyl chitosan/aldehyde-hyaluronic acid	Mice	NR	Day 3: >50%; Day 10: nearly complete healing	NR	NR	NR	Stimulation of cell proliferation, migration, and endothelial tube formation while suppressing macrophage-derived pro-inflammatory signals (Zhang S. et al., 2026)
Full-thickness skin defect	Dental pulp stem cells	GelMA	Mice	NR	Day 9: >90% (Gel-EVs); 71% (GelMA); 86% (EVs)	NR	NR	NR	MiR-486-5p EVs improved angiogenic activities of HUVECs by increasing the TGF-β1 expression and Smad2 phosphorylation, while decreasing the Smurf2 expression (Wang S. et al., 2026)
Full-thickness skin defect	Paris polyphylla var. yunnanensis	Polyvinyl pyrrolidone/carboxymethyl chitosan	Mice	NR	Day 7: 82.17% ± 5.44%; Day 14: 99.66% ± 0.42%	NR	NR	IL-1β, IL-6 and TNF-α: >1.5-fold decreased vs. model group; intracellular ROS: 50% decreased in high-concentration PPEV group vs. positive control	Reduced the expression of pro-inflammatory cytokines (Tang et al., 2025)
Full-thickness skin defect	Periosteum	NAGA/GelMA/Laponite/glycerol	Rats	NR	Day 14: significantly increased	NR	NR	NR	Enhanced the proliferation, migration, and tube formation of ECs *in vitro* (Han et al., 2022)
Diabetic wound	ADSCs	Epsilon-poly-lysine and hyaluronic acid	Mice	NR	Day 15: significantly accelerated	NR	NR	NR	Increased angiogenesis, increased collagen deposition, enhanced cell proliferation, reduced inflammation, and improved tissue architecture (Ju et al., 2025)
Diabetic wound	UCMSCs	GelMA	Rats	NR	Day 14: significantly accelerated	NR	NR	NR	Promoted HUVEC cell migration; increased the wound collagen content and re-epithelialization (Tang et al., 2023)
Diabetic wound	Platelet-rich plasma	Gelatin/β-cyclodextrin	Rats	NR	Day 14: significantly accelerated	NR	NR	NR	Activated of autophagy and inhibition of apoptosis in HUVECs and HSFs, induced the blood vessel creation, collagen formation and re-epithelialization (Shu et al., 2023)
Diabetic wound	ADSCs	Extracellular matrix hydrogel	Mice	200 μg/mL	Day 14: 92% (ECM@Exo) vs. 50% (Control)	NR	NR	NR	Reduced inflammation and promotes angiogenesis, collagen deposition, cell proliferation, and migration (Song et al., 2023)
Diabetic wound	MSCs	Gelatin methacryloyl (Gel)/poly-L-lysine methacryloyl (PL)-5 hydrogel (Gel/PL-5)	Mice	NR	Day 14: significantly accelerated	NR	NR	NR	Stimulated HUVECs; exhibited angiogenesis and anti-inflammation (Wang et al., 2024)
Diabetic wound	Houttuynia cordata thunb	rhCOL3/3-APBA	Mice	NR	Significantly accelerated	NR	NR	NR	Improved angiogenesis, increased collagen deposition, and reduced inflammation; regulating both NF-κB and YAP pathways (Yang W. et al., 2025)
Diabetic wound	ADSCs	HAP/OCS/PEG/Ag-E	Mice	NR	Day 10: near-complete closure	NR	NR	NR	Promoted M2 macrophage polarization, reduced pro-inflammatory cytokines (IL-1β, IL-6, TNF-α), and accelerated wound healing with enhanced angiogenesis and collagen deposition; suppressed the Notch/NF-κB/NLRP3 signaling pathway (Zhang Y. et al., 2025)
Chronic inflammation wound	MSCs	CMCS/OHA	Mice	NR	Day 14: significantly accelerated	NR	NR	NR	Inhibited the secretion of inflammatory factors; promoted wound re-epithelization and collagen production (Yue et al., 2024)
Burn wound	ADSCs	ADA-aPF127	Rats	NR	Day 21: significantly accelerated	NR	NR	NR	Accelerated epithelialization, promoting granulation tissue formation and collagen deposition, hair follicle regeneration, mitigating inflammatory responses, and facilitating enhanced neovascularization (Vipin and Kumar, 2025)
MRSA- infected wound	MSCs	pH/ROS-responsive hydrogel (GA-containing)	Mice	NR	Significantly accelerated	NR	NR	NR	Promoted angiogenesis, promoted epithelialization, antibacterial against MRSA (Chen X. et al., 2025)
MRSA-infected wound	Broccoli	Silk protein methacryloyl hydrogel	Mice	NR	Day 7: 76.89% ± 1.70%; Day 14: 99.29% ± 0.71%	NR	NR	NR	Improved angiogenesis, increased collagen deposition, NF-κB inhibition and M2 macrophage polarization (Duan et al., 2025)

NR, Not reported. Quantitative values were included only when numerical data were explicitly reported in the original study; outcomes presented only qualitatively or graphically without directly extractable numerical values were recorded as NR.

### 7.1 Acute skin wounds

Acute wounds result from external forces such as lacerations, abrasions, and contusions and can heal spontaneously within 1–2 weeks under normal physiological conditions, with core therapeutic requirements of rapid hemostasis, anti-infection, and initiation of re-epithelialization. Given the high mobility and short healing window of acute wounds, the design of hydrogel-EV systems prioritizes strong tissue adhesion, rapid hemostatic capacity, and early burst release of EVs to match the healing timeline. Sun et al. addressed this requirement with a tough, highly adhesive hydrogel (adhesive strength reaching 200 kPa) whose stable network enabled rapid hemostasis in acute wounds (Sun et al., 2025). Beyond achieving swift bleeding control, such high-strength adhesives also resist the tensile forces that challenge wound closure under dynamic conditions (Song et al., 2026).

For re-epithelialization promotion, SerMA hydrogel loaded with royal jelly-derived EVs achieved promoted re-epithelialization in a full-thickness skin defect model through Wnt signaling pathway activation and inflammation suppression (Tan et al., 2023). Additionally, hydrogel-EV systems promote keratinocyte proliferation and migration and drive wound angiogenesis, constituting multi-faceted effects for acute wound healing (Shaw et al., 2025; Wang S. et al., 2026; Zhang S. et al., 2026). In essence, the main requirement for hydrogel-EV systems in acute wounds is rapid response, whereby the material’s high adhesion and rapid hemostasis provide a stable local action window for EVs, and early EV release rapidly initiates the epithelial regeneration and angiogenic cascade.

### 7.2 Diabetic chronic non-healing wounds

Diabetic chronic wounds represent one of the most severe complications of diabetes, characterized by healing that extends beyond 4 weeks, recurrent infection, impaired angiogenesis, and defective epithelialization. Underlying these features is a vicious cycle driven by hyperglycemia, in which metabolic dysregulation, immune dysfunction, and microcirculatory disturbance perpetuate wound stagnation. Hydrogel-EV systems can intervene at multiple nodes within this pathological cascade and have received increasing research attention in this field (Liu J. et al., 2022; Wang et al., 2024; Tsai et al., 2025; Zhou et al., 2025).

A principal therapeutic strategy for managing chronic diabetic wounds is to disrupt the self-reinforcing loop within the microenvironment. The ADSC-EV-loaded ε-polylysine/hyaluronic acid dual-crosslinked antibacterial hydrogel exemplifies this strategy, improving wound tissue architecture in diabetic mice through concurrent infection control, angiogenesis promotion, and inflammation alleviation (Ju et al., 2025). The ECM@exo system similarly enhanced collagen deposition and cell proliferation in diabetic wounds (Song et al., 2023). Meanwhile, the rhCOL3/APBA-responsive hydrogel loaded with HC-EVs simultaneously regulated the NF-κB and YAP pathways, concurrently modulating inflammatory and proliferative pathways (Yang W. et al., 2025).

Stimuli-responsive hydrogels have proven therapeutic potential in diabetic wound applications by converting the wound’s endogenous pathological signals into triggers for microenvironment-responsive EV release. These microenvironment-responsive systems adapt their release kinetics to the evolving biochemical milieu across successive healing stages, aligning therapeutic intervention with pathological progression. For instance, carboxymethyl alginate/oxidized hyaluronic acid hydrogels form rapidly *in situ* through Schiff-base reactions and absorb excess glucose from the wound environment; the absorbed glucose, in turn, attenuates the competitive effect of borax on Schiff-base bonds, thereby reinforcing the sustained adhesive capacity of the hydrogel at the wound site (Li W. X. et al., 2026). In another approach, the dual-responsive GPP@ZnBG hydrogel responds to both elevated glucose and oxidative stress in diabetic wounds, releasing therapeutic metal ions in a self-regulated, sequential manner to promote repair (Zhao et al., 2026). These examples complement earlier glucose/pH dual-responsive systems such as HAP/OCS/PEG/Ag-E and MMP-9 enzyme-responsive strategies, collectively illustrating the progression from single-stimulus to multi-stimulus responsive designs (Meng et al., 2025; Zhang Y. et al., 2025).

Unlike acute wounds, diabetic chronic wounds demand sustained regulation, requiring not only prolonged EV supply over a therapeutic window of weeks to months but also responsiveness to changes in the dynamically changing wound microenvironment. This requirement has prompted the progression of dual-responsive and multi-responsive systems from proof-of-concept validation toward preclinical evaluation.

### 7.3 Burn wounds

Burns are a special category of skin injury caused by high temperature, electric current, chemical agents, or radiation, classified by depth into first-to third-degree burns. Their healing is complicated by intense inflammation, oxidative stress, a high risk of infection, slow epithelial regeneration, and a pronounced tendency toward hypertrophic scarring. Hydrogel-EV systems designed for burn repair must therefore integrate antibacterial, anti-inflammatory, pro-regenerative, and scar-mitigating functions within a single platform. Vipin et al. demonstrated one such approach with a sprayable thermosensitive polysaccharide-based hydrogel (ADA-aPF127@LL18/Exo) that combined sustained EV release with antibacterial activity, accelerating epithelialization and collagen deposition in a rat burn model (Vipin and Kumar, 2025). Sprayable formulations may be useful in burn care, as they enable delivery without direct wound contact. Compared with acute and diabetic wounds, burn wound repair demands more complex multi-objective optimization, and stratified delivery strategies tailored to burn depth and surface area remain largely unexplored.

### 7.4 Other wound types

Beyond acute, diabetic, and burn wounds, the application of EV-loaded hydrogels in other wound types remains relatively limited. One study reported that a self-adaptive multifunctional hydrogel loaded with MSC-EVs accelerated healing of full-thickness skin wounds infected with methicillin-resistant *Staphylococcus aureus* (MRSA) in mice (Chen X. et al., 2025). Another study concerning infected wound healing adopted silk protein methacryloyl hydrogel as a carrier loaded with broccoli-derived EVs and epigallocatechin gallate-gold nanoparticles. This composite system facilitated the repair of MRSA-infected wounds in mice via multifaceted mechanisms, such as eliminating MRSA pathogens and mitigating the inflammatory microenvironment (Duan et al., 2025). In contrast, evidence for postoperative wounds, pressure ulcers, venous leg ulcers, ischemic wounds, and radiation-induced wounds is still scarce. Consequently, expanding EV-loaded hydrogel platforms to these clinically challenging wounds represents an important direction for future research.

In summary, this pathology-guided differentiated design strategy may provide a useful approach for advancing hydrogel-EV systems from generic dressings toward pathology-oriented wound repair strategies. Notably, although almost all published preclinical studies have reported beneficial effects of hydrogel-EV systems, several limitations should be acknowledged. Most evidence is derived from small-animal models with relatively short observation periods and limited sample sizes. Direct comparisons among different EV sources, hydrogel formulations, and loading strategies remain scarce, making it difficult to determine the optimal therapeutic hydrogel-EV system.

### 7.5 Evidence levels and limitations of hydrogel-EV systems for wound healing

The current evidence for hydrogel-EV systems spans multiple levels of experimental validation (Table 6) but remains predominantly based on small-animal studies. Large-animal validation is scarce, and direct human evidence remains lacking, leaving a substantial gap between preclinical efficacy and clinical translation. In addition, heterogeneity in EV preparation, hydrogel formulation, dosing regimens, experimental models, and outcome assessment limits cross-study comparability. Future studies should therefore prioritize methodological standardization, validation in clinically relevant large-animal models, and subsequent well-designed human studies to establish reproducibility, safety, and therapeutic efficacy.

**TABLE 6:** Evidence levels and critical appraisal of hydrogel-EV systems for skin wound healing.

Highest evidence level	Representative studies	Wound/model types	Main findings	Major limitations
*In vitro*	(De Cesare et al., 2026; Nurfajrin et al., 2026)	Fibroblast/MSC-related skin-repair assays; human 1BR3 skin fibroblast model	EV-loaded hydrogels showed favorable cytocompatibility and promoted wound-relevant cellular responses, including fibroblast activity, migration/proliferation, and regenerative responses	Simplified cell-culture systems cannot reproduce the inflammatory, vascular, immune, metabolic, and mechanical complexity of wounds and provide no direct evidence regarding *in vivo* retention, biodistribution, systemic safety, or whole-wound efficacy
Small animal	(Li et al., 2021; Han et al., 2022; Song et al., 2023; Tang et al., 2023; Wang et al., 2023; Liu S. et al., 2024; Wang et al., 2024; Liu B. et al., 2025; Meng et al., 2025; Vipin and Kumar, 2025; Zhang Y. et al., 2025; Subhan et al., 2026; Taghdi et al., 2026; Wang S. et al., 2026; Yang et al., 2026)	Predominantly mouse and rat models of full-thickness/acute, diabetic, infected, chronic inflammatory, and burn wounds	Collectively, EV-hydrogel systems improved wound closure and re-epithelialization, angiogenesis, collagen deposition/remodeling, and inflammatory regulation. Recent studies additionally evaluated controlled EV release, local retention/biodistribution, and defined molecular mechanisms	Evidence remains dominated by rodents. Rodent skin healing relies more strongly on contraction than human healing. Marked heterogeneity in EV source/preparation, hydrogel composition, EV dose, treatment regimen, wound model, follow-up duration, and outcome measures limits cross-study comparison and clinical extrapolation
Large animal	(Cui et al., 2026)	Porcine full-thickness skin excision model	Chitosan hydrogel combined with MSC-derived exosomes accelerated wound closure and epithelialization, reduced wound contraction and inflammation, enhanced angiogenesis and type I collagen deposition, and produced more mature skin architecture	Large-animal evidence remains extremely sparse. Independent replication, standardized dosing, EV release/retention, long-term safety remain insufficient
Human	Direct clinical hydrogel-EV wound-healing evidence not identified	Not applicable	Not applicable	Clinical efficacy, optimal EV dose, hydrogel formulation, treatment frequency, manufacturing consistency, long-term safety, and clinically meaningful endpoints remain to be established in prospective controlled human studies

Studies were classified according to the highest level of experimental validation. Studies containing both *in vitro* and small-animal experiments were classified as small-animal evidence and were not duplicated *in vitro* evidence.

## 8 Discussion

Although hydrogel-EV systems have accumulated substantial preclinical evidence for wound repair, their clinical translation still faces multiple challenges concerning production standardization, system construction optimization, large-scale manufacturing, and regulatory pathways (Figure 6).

**FIGURE 6:**
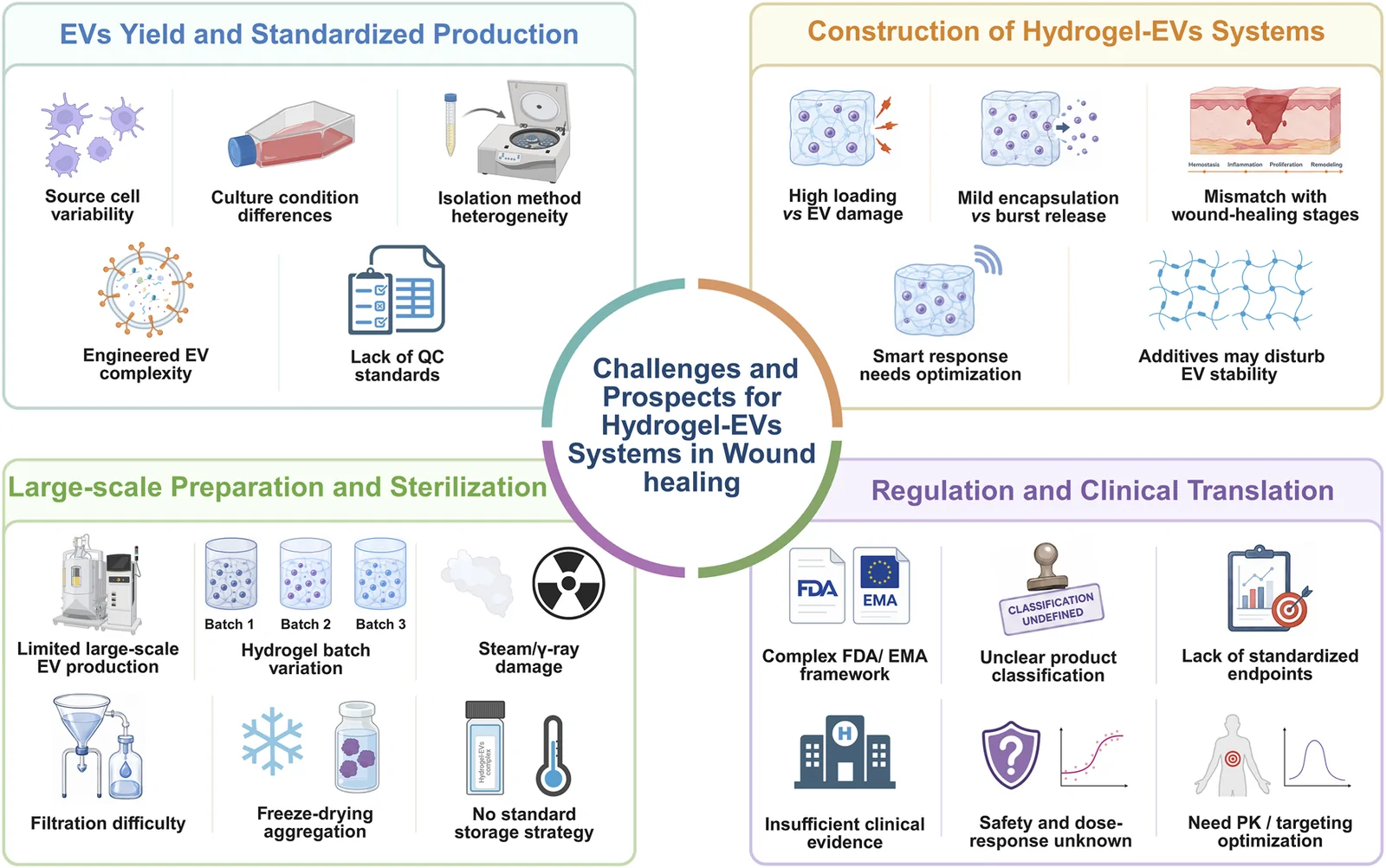
Current challenges and future perspectives for hydrogel-EV systems in wound healing. Key barriers to clinical translation include limited EV yield and batch-to-batch consistency, incomplete standardization of EV isolation and quality control, challenges in EV-hydrogel formulation and activity preservation, scale-up and sterilization requirements, and unresolved regulatory and clinical issues. Translation toward clinical application requires standardized manufacturing, validated quality-control and release criteria, systematic safety assessment, and evaluation of pharmacokinetics and biodistribution. EVs, extracellular vesicles; CMC, chemistry, manufacturing, and controls; QC, quality control; PK, pharmacokinetics; FDA, U.S. Food and Drug Administration; EMA, European Medicines Agency.

The primary bottleneck lies in the low yield and the absence of standardized production of EVs. Most current studies rely on MSCs, immune cells, or plant-derived cells for EV extraction, yet variations in donor cells, culture conditions, and isolation methods lead to marked heterogeneity in particle size distribution, surface marker expression, and functional activity (Hade et al., 2022). Although engineering modifications can enhance targeting capability and drug-loading efficiency, they further increase manufacturing complexity (Liao et al., 2024). To date, internationally recognized quality control standards for EVs, covering purity, concentration, functional activity metrics, and batch-to-batch consistency, remain absent, severely hampering large-scale production and consistent clinical evaluation (Le et al., 2025).

Second, the construction of hydrogel-EV systems faces several inherent conflicts. One conflict is the trade-off between loading efficiency and EV bioactivity, where strong interactions such as covalent immobilization or high-concentration mixing can increase loading but may damage the EV membrane structure and compromise bioactivity, whereas gentle physical encapsulation favors activity retention yet often leads to burst release or rapid loss (Chen Y. et al., 2023; Li Z. et al., 2024; Zheng et al., 2024). Another conflict arises between multifunctional integration and component compatibility. When additional functions such as antibacterial and antioxidant properties are imparted, components like silver nanoparticles, cationic polymers, or tea tree oil may interfere with EV membrane stability or inhibit the function of encapsulated miRNAs (Ju et al., 2025). For example, an ε-polylysine-hyaluronic acid system combines antibacterial activity with EV delivery, but the potential impact of high concentrations of cationic polymers on EV integrity has not been fully evaluated. A third conflict involves matching release kinetics with the four phases of wound healing, namely hemostasis, inflammation, proliferation, and remodeling. Although stimuli-responsive hydrogels can achieve on-demand release, systematic validation of their sensitivity, specificity, and the improved temporal alignment of release kinetics with each phase is still lacking, which represents a critical bottleneck for the clinical translation of stimuli-responsive hydrogels (Ding K. et al., 2025).

Furthermore, large-scale manufacturing and sterilization procedures constitute engineering hurdles. EV mass production is constrained by cell expansion efficiency and vesicle purification costs. Conventional culture systems can yield only a limited number of doses, whereas the introduction of bioreactors and hollow-fiber culture systems holds promise for significantly increasing production capacity (Zheng et al., 2024). Hydrogel batch-to-batch consistency is also markedly influenced by raw material sources, such as animal-derived batch variations of GelMA, and crosslinking conditions including light intensity, temperature, and catalyst residues. Sterilization also poses challenges to the stability of the composite system. Traditional autoclaving or γ-irradiation may cause EV protein denaturation, lipid membrane rupture, and nucleic acid degradation, while filter sterilization is limited by the overlap between EV particle size and typical filter pore sizes of 0.22 μm. Although lyophilization with protective agents such as trehalose and mannitol can improve storage stability, the lyophilization–reconstitution process may still induce EV aggregation or activity loss (Galbiati et al., 2025). Currently, no standardized good manufacturing practice (GMP)-compliant manufacturing process exists for hydrogel-EV systems. Critical issues, including aseptic manufacturing, process validation, chemistry, manufacturing and controls (CMC), product release criteria, and long-term preservation protocols, remain insufficiently established, creating major engineering barriers to industrial production and clinical application. In addition, ambiguous regulatory frameworks and a paucity of clinical evidence impede the translational process.

Regulatory agencies such as the United States Food and Drug Administration (FDA) and the European Medicines Agency (EMA) require clear definition of the cell source, manufacturing process, quality attributes, and mechanism of action of EVs, yet EVs as multicomponent bioactive entities are neither small-molecule drugs nor conventional biological products, which poses unique challenges for pharmacological quality assessment and potency determination. Preclinical studies have validated the efficacy of various composite systems in diabetic, aged, and full-thickness skin defect models, but standardized efficacy endpoints, such as healing rate, scar score, and functional recovery indicators, are lacking (Yang Y. et al., 2025). Human evidence is limited to one preliminary study demonstrating a positive correlation between wound fluid microvesicle concentration and healing rate through nanoparticle tracking analysis, while data on safety, dose-response relationships, and long-term follow-up for composite products remain absent (Mari et al., 2024; Stiliyanov-Atanasov et al., 2025). In addition, the long-term safety of multifunctional hydrogel-EV systems remains insufficiently characterized. Potential concerns include immunogenicity, off-target effects, abnormal angiogenesis, pro-fibrotic responses, residual contaminants introduced during EV isolation or hydrogel fabrication, toxicity associated with nanomaterials, and biosafety issues related to genetically engineered EVs. These aspects require systematic toxicological evaluation before clinical application.

To address these bottlenecks, future research should concentrate on several directions. First, the standardization and automation of EV production should be advanced by establishing comprehensive standard operating procedures covering donor cell qualification, culture conditions, isolation and purification process validation, GMP-compliant manufacturing, CMC documentation, aseptic processing, product release criteria, with international societies such as the International Society for Extracellular Vesicles (ISEV) taking the lead in formulating specific guidelines for wound repair. Concurrently, 3D bioreactors, hollow-fiber systems, and continuous-flow purification technologies should be introduced to achieve a stable and scalable supply of EVs. Second, integrated multifunctional hydrogel platforms need to be developed that combine antibacterial, antioxidant, oxygen-releasing, and stimuli-responsive capabilities with EV delivery within a single system, while systematically evaluating the compatibility of each functional component with EVs to overcome the conflict between loading efficiency and activity retention. Third, pathology-oriented design strategies should be implemented, where EV sources, engineering strategies, and release kinetics are customized based on the molecular characteristics of different wound types and healing stages, with a strengthened emphasis on *in vivo* release validation of smart hydrogels. Fourth, bridging the gap between preclinical and clinical studies requires standardized quality evaluation of EV-hydrogel products, including identity, purity, potency, and stability. Pharmacokinetic and biodistribution studies are needed to characterize the *in vivo* fate of EVs after release from hydrogel matrices. Subsequent dose-escalation clinical studies should evaluate safety and effective dose ranges, accompanied by regulatory efforts to establish appropriate pathways for EV-biomaterial combination products.

In summary, hydrogel-mediated EV delivery for skin wound repair has progressed from proof-of-concept studies toward broader preclinical development and evaluation. This review has examined EV source diversity and engineering strategies, hydrogel design principles and stimuli-responsive systems, modes of EV-hydrogel integration, and the biological processes involved in wound repair. These processes, including inflammatory regulation, angiogenesis, ECM remodeling, and cell proliferation and migration, are interconnected, while the hydrogel matrix additionally contributes to EV retention, release, and local microenvironmental regulation. At the application level, pathology-guided design strategies have promoted the evolution from conventional wound dressings toward more adaptive and individualized therapeutic platforms. Nevertheless, several key challenges remain unresolved. Current evidence is derived predominantly from *in vitro* and small-animal studies, while large-animal studies and clinical validation remain scarce. In addition, the heterogeneity of EV sources, the lack of standardized manufacturing and quality control, incomplete understanding of long-term safety, and the absence of unified regulatory standards continue to hinder clinical translation. Further progress toward clinical translation will require standardized EV production, GMP-compliant manufacturing, comprehensive safety evaluation, and clearer regulatory frameworks for EV-hydrogel products.
