# Supplementary material for: Hydrogel-based delivery of extracellular vesicles for skin wound healing: design strategies, therapeutic mechanisms, and translational perspectives
Source: Front Bioeng Biotechnol. 2026 Oct 16;14:1976532. doi: 10.3389/fbioe.2026.1976532 (PMC13720249; doi:10.3389/fbioe.2026.1976532)
Supplement: Supplementary file 1 [file Supplementaryfile1.docx]

**Literature Search and Study Selection**

A structured literature search was conducted in PubMed and the Web of Science Core Collection (WoSCC) from database inception to September 25, 2026. The search strategy combined terms related to extracellular vesicles (“extracellular vesicle*” OR “exosome*”), hydrogels (“hydrogel*”), and skin wound healing (“skin wound*,” “wound healing,” “diabetic wound*,” “chronic wound*,” “infected wound*,” “burn wound*,” “surgical wound*,” and “skin regeneration”). Boolean operators (AND/OR) were used to combine search concepts, and database-specific search syntax was applied. The complete search strategies for each database are provided in Supplementary material.

Original experimental studies were considered core evidence when they investigated EVs or clearly defined EV-related vesicular systems incorporated into or delivered by a hydrogel and evaluated the resulting system in the context of cutaneous wound healing or skin regeneration. Eligible studies could include in vitro, small-animal, large-animal, or human investigations, provided that relevant wound-healing or skin-regeneration outcomes were assessed and sufficient methodological information was available. Studies were excluded from the core evidence set if they did not investigate an EV-based vesicular component, did not involve a hydrogel delivery system, were unrelated to cutaneous wound healing or skin regeneration, were non-original publications, were duplicate reports, or provided insufficient information for assessment.

The searches identified 441 records from PubMed and 724 records from WoSCC. After removal of 398 duplicates, 767 unique records remained for title and abstract screening. Of these, 248 potentially eligible articles underwent full-text assessment, and 95 studies meeting the predefined eligibility criteria were retained as the core evidence set for hydrogel-EV systems in cutaneous wound healing. Because this narrative review also with a broader mechanistic and translational scope, additional references were used where necessary to support background topics, including EV characterization and quality control, EV engineering, hydrogel design principles, wound pathophysiology, stimuli-responsive biomaterials, manufacturing, safety, and regulatory considerations. Additional references were identified through targeted topic-specific searches and manual screening of reference lists.

**Database: Pubmed:**

#1 "extracellular vesicle*"[Title/Abstract] OR "exosome*"[Title/Abstract]

#2 "hydrogel*"[Title/Abstract]

#3 "skin wound*"[Title/Abstract] OR "wound healing"[Title/Abstract] OR "diabetic wound*"[Title/Abstract] OR "chronic wound*"[Title/Abstract] OR "infected wound*"[Title/Abstract] OR "burn wound*"[Title/Abstract] OR "surgical wound*"[Title/Abstract] OR "skin regeneration"[Title/Abstract]

#1 AND #2 AND #3

Timespan: From database inception to September 25, 2026

**Database: Web of Science Core Collection (WoSCC):**

Search stragegy:

#1 TS= ("extracellular vesicle*" OR exosome*)

#2 TS= (hydrogel*)

#3 TS= ("skin wound*" OR "wound healing" OR "diabetic wound*" OR "infected wound*" OR "burn wound*" OR "surgical wound*" OR "skin regeneration" OR "chronic wound*")

#1 AND #2 AND #3

Timespan: From database inception to September 25, 2026
